# Allogenic Umbilical Cord-Derived Mesenchymal Stromal Cells Sustain Long-Term Therapeutic Efficacy Compared With Low-Dose Interleukin-2 in Systemic Lupus Erythematosus

**DOI:** 10.1093/stcltm/szad032

**Published:** 2023-06-06

**Authors:** Zhouli Cao, DanDan Wang, Lijuan Jing, Xin Wen, Nan Xia, Wenjuan Ma, Xueyi Zhang, Ziyi Jin, Wei Shen, Genhong Yao, Weiwei Chen, Xiaojun Tang, Linyu Geng, Hui Li, Xiaojing Li, Shuai Ding, Jun Liang, Xuebing Feng, Huayong Zhang, Shanshan Liu, Wenchao Li, Lingyun Sun

**Affiliations:** Department of Rheumatology and Immunology, The Affiliated Drum Tower Hospital of Nanjing University Medical School, Nanjing, Jiangsu, People’s Republic of China; Department of Rheumatology and Immunology, The Affiliated Drum Tower Hospital of Nanjing University Medical School, Nanjing, Jiangsu, People’s Republic of China; Department of Rheumatology and Immunology, The Affiliated Drum Tower Hospital of Nanjing University Medical School, Nanjing, Jiangsu, People’s Republic of China; Department of Rheumatology and Immunology, The Affiliated Drum Tower Hospital of Nanjing University Medical School, Nanjing, Jiangsu, People’s Republic of China; Department of Rheumatology and Immunology, The Affiliated Drum Tower Hospital of Nanjing University Medical School, Nanjing, Jiangsu, People’s Republic of China; Department of Rheumatology and Immunology, The Affiliated Drum Tower Hospital of Nanjing University Medical School, Nanjing, Jiangsu, People’s Republic of China; Nanjing Drum Tower Hospital of China Pharmaceutical University, Jiangsu, Nanjing, Jiangsu, People’s Republic of China; Department of Rheumatology and Immunology, The Affiliated Drum Tower Hospital of Nanjing University Medical School, Nanjing, Jiangsu, People’s Republic of China; Nanjing Drum Tower Hospital of China Pharmaceutical University, Jiangsu, Nanjing, Jiangsu, People’s Republic of China; Nanjing Drum Tower Hospital of China Pharmaceutical University, Jiangsu, Nanjing, Jiangsu, People’s Republic of China; Department of Rheumatology and Immunology, The Affiliated Drum Tower Hospital of Nanjing University Medical School, Nanjing, Jiangsu, People’s Republic of China; Department of Rheumatology and Immunology, The Affiliated Drum Tower Hospital of Nanjing University Medical School, Nanjing, Jiangsu, People’s Republic of China; Department of Rheumatology and Immunology, The Affiliated Drum Tower Hospital of Nanjing University Medical School, Nanjing, Jiangsu, People’s Republic of China; Department of Rheumatology and Immunology, The Affiliated Drum Tower Hospital of Nanjing University Medical School, Nanjing, Jiangsu, People’s Republic of China; Department of Rheumatology and Immunology, The Affiliated Drum Tower Hospital of Nanjing University Medical School, Nanjing, Jiangsu, People’s Republic of China; Department of Rheumatology and Immunology, The Affiliated Drum Tower Hospital of Nanjing University Medical School, Nanjing, Jiangsu, People’s Republic of China; Department of Rheumatology and Immunology, The Affiliated Drum Tower Hospital of Nanjing University Medical School, Nanjing, Jiangsu, People’s Republic of China; Department of Rheumatology and Immunology, The Affiliated Drum Tower Hospital of Nanjing University Medical School, Nanjing, Jiangsu, People’s Republic of China; Department of Rheumatology and Immunology, The Affiliated Drum Tower Hospital of Nanjing University Medical School, Nanjing, Jiangsu, People’s Republic of China; Department of Rheumatology and Immunology, The Affiliated Drum Tower Hospital of Nanjing University Medical School, Nanjing, Jiangsu, People’s Republic of China; Department of Rheumatology and Immunology, The Affiliated Drum Tower Hospital of Nanjing University Medical School, Nanjing, Jiangsu, People’s Republic of China; Department of Rheumatology and Immunology, The Affiliated Drum Tower Hospital of Nanjing University Medical School, Nanjing, Jiangsu, People’s Republic of China; Department of Rheumatology and Immunology, The Affiliated Drum Tower Hospital of Nanjing University Medical School, Nanjing, Jiangsu, People’s Republic of China; Department of Rheumatology and Immunology, The Affiliated Drum Tower Hospital of Nanjing University Medical School, Nanjing, Jiangsu, People’s Republic of China; Nanjing Drum Tower Hospital of China Pharmaceutical University, Jiangsu, Nanjing, Jiangsu, People’s Republic of China; Department of Rheumatology and Immunology, The First Affiliated Hospital of Anhui Medical University, Hefei, People’s Republic of China

**Keywords:** mesenchymal stromal cells, interleukin-2, regulatory T cells, systemic lupus erythematosus

## Abstract

**Objectives:**

Mesenchymal stromal cells (MSCs) and low-dose interleukin-2 (IL-2) both have demonstrated efficacy in treating systemic lupus erythematosus (SLE). The aim of this study is to conduct a head-to-head comparison between the 2 treatments and provide insights for clinical applications.

**Methods:**

Lupus-prone mice were treated with umbilical cord-derived MSCs (UC-MSCs), IL-2, or a combination of UC-MSCs and IL-2, respectively. The lupus-like symptoms, renal pathology, and T-cell response were assessed 1 or 4 weeks later. Modulation of IL-2 production by MSCs on immune cells was investigated by the coculture assay. Disease activity and serum IL-2 of SLE patients were determined before and after receiving UC-MSCs.

**Results:**

Both UC-MSCs and IL-2 improved lupus symptoms in lupus-prone mice 1 week after treatment, while the effects of UC-MSCs lasted up to 4 weeks. Moreover, the UC-MSC-treated group showed better renal pathology improvement. Importantly, UC-MSCs combined with IL-2 did not provide better efficacy than UC-MSCs alone. Consistent with this, UC-MSCs alone and UC-MSCs + IL-2 resulted in similar levels of serum IL-2 and frequencies of Tregs. Neutralization of IL-2 partly reduced the promotion of Tregs by UC-MSCs, suggesting that IL-2 was involved in the upregulation of Tregs by UC-MSCs. Lastly, an increase in serum IL-2 positively correlated with the reduction of disease activity of SLE patients by UC-MSCs.

**Conclusion:**

Both the single injection of UC-MSCs and repeated IL-2 administration exerted comparable efficacy in alleviating SLE manifestations, but UC-MSCs provided sustained alleviation and showed better improvement in renal pathology.

## Graphical Abstract

**Graphical Abstract:**
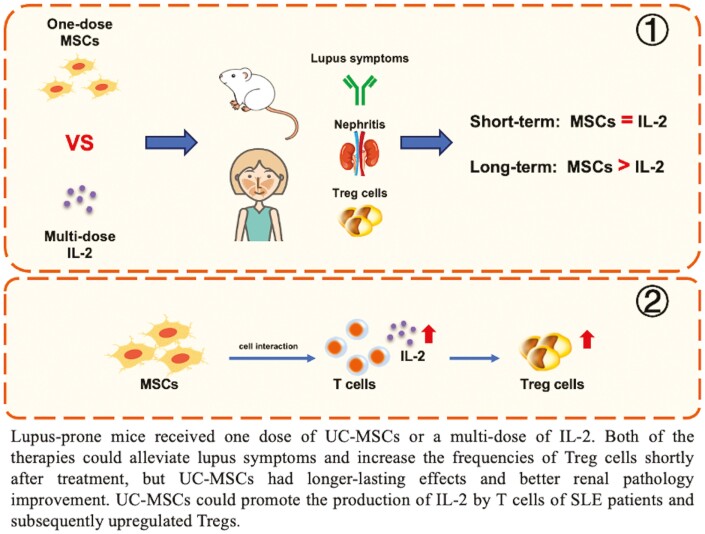

**Significance Statement**

This study found that repeated IL-2 administration and one single injection of UC-MSCs are comparable in upregulating serum IL-2 and alleviating SLE manifestations short after treatment. One single injection of UC-MSCs provides sustained alleviation of SLE manifestations, especially in renal pathology, compared with repeated IL-2 administration. Therefore, UC-MSCs alleviate SLE manifestations through sustained upregulation of serum IL-2, overcoming the disadvantage of repeated IL-2 administration.

## Introduction

Systemic lupus erythematosus (SLE) is a quintessential multisystem autoimmune disease characterized by abnormal activation of T and B lymphocyte.^1,2^ The current application of glucocorticoids and immunosuppressive agents has a high non-response rate and several unfavorable drawbacks, including liver damage, bone marrow suppression, and fundus disease. In recent years, mesenchymal stromal cells (MSCs) have become an attractive choice for immune disorder treatments.^3^ MSCs are multifunctional stromal cells derived from the early mesoderm and have a high degree of immunoregulatory potential.^4^ According to the published clinical trials in the last decades, MSCs treatment has shown significant efficacy and excellent safety in treating SLE.^5-7^ The total response rate of refractory lupus nephritis patients receiving allogeneic umbilical cord mesenchymal stromal cells (UC-MSCs) was 60%, and the mortality rate of 2-5 years decreased from 35%-45% to 6%.^8^ MSCs modulate the immune system of SLE patients either by secreting soluble factors or directly interacting with a variety of immune effector cells.^3,9^

Regulatory T cells (Tregs) are a subset of CD4^+^ T cells required for self-tolerance maintenance by suppressing autoreactive lymphocytes. Defects in Tregs have been considered important aspects of SLE pathogenesis.^10^ Accumulating evidence has confirmed the role of UC-MSCs in promoting the expansion and immune-suppressive potency of Tregs, which thereby strongly inhibit inflammatory responses.^11,12^ Although the mechanism of Treg induction by MSCs is still not completely understood, it is well-known that both soluble factors and cell-contact-dependent events are involved. In the murine colitis model, MSCs isolated from human adipose were reported to activate regulatory T cells by cell–cell contact and prostaglandin E2 (PGE2) production.^13^ While in the context of acute graft versus host disease (GvHD), human UC-MSCs (hUC-MSCs) transferred mitochondria to CD4^+^T cells and increased the expression of genes involved in T-cell activation and Treg differentiation, including fork head box P3 (FOXP3), interleukin-2 receptor alpha chain (IL2RA), cytotoxic T-lymphocyte associated protein 4 (CTLA4), and transforming growth factor β(TGF-β), and led to an increase in a highly suppressive T-regulatory-cell population.^14^ Moreover, studies have shown that human bone marrow derived-MSCs (hBM-MSCs) can interact with Tregs through specific receptors, such as insulin-like growth factor binding protein-4 (IGFBP-4), and promote their survival, migration, and expansion.^15^ Recently, Yang et al. showed that UC-MSCs could modulate the gut microbiome and influence the gut-immune axis and lead to the induction of Tregs.^16^ Together, the modulating mechanisms of Tregs by MSCs may vary depending on the source, the dose, and the context of administration, as well as the model used. To fully understand the immuno-regulating mechanisms of MSCs and the optimal conditions for their use, further investigation is needed.

IL-2, primarily produced by activated T cells, is essential for the thymic development and differentiation of Tregs and their growth and survival in peripheral tissue.^17^ Unlike effector T cells (Teffs), Tregs constitutively express high levels of the IL-2 receptor, CD25, and respond to low concentrations of IL-2.^18^ Taking advantage of this property, several clinical trials reported that low-dose IL-2 exerted a selective effect on Tregs in patients with type 1 diabetes, GvHD, and alopecia areata. Significantly impaired IL-2 production has been observed in T cells from both SLE patients and lupus mice. Moreover, IL-2 deficiency is positively correlated with disease progression and immunopathology,^19,20^ suggesting that IL-2 restoration could be a possible solution to SLE.^21-23^ Recently, a randomized controlled clinical trial that recruited 60 SLE patients confirmed that exogenous IL-2 directly expanded the Tregs population, exhibiting great clinical efficacy on active and refractory SLE.^21^

Considering that both MSCs and IL-2 shared similarities in their therapeutic effects by increasing Tregs, several important questions are raised and should be addressed. First, what are the differences between the 2 therapies in controlling SLE progression; second, whether IL-2 is involved in the MSCs-induced increase of Tregs in treating lupus; lastly, would IL-2 and MSCs together exert better therapeutic effects? Clarifying these questions will not only deepen understanding of the therapeutic mechanisms of MSCs and IL-2 on SLE but also provide great help to the clinical treatment of SLE and other immune disorders.

## Result

### UC-MSCs and Low-Dose IL-2 Treatment Alleviated Lupus Diseases in Mice

**Figure 1.**
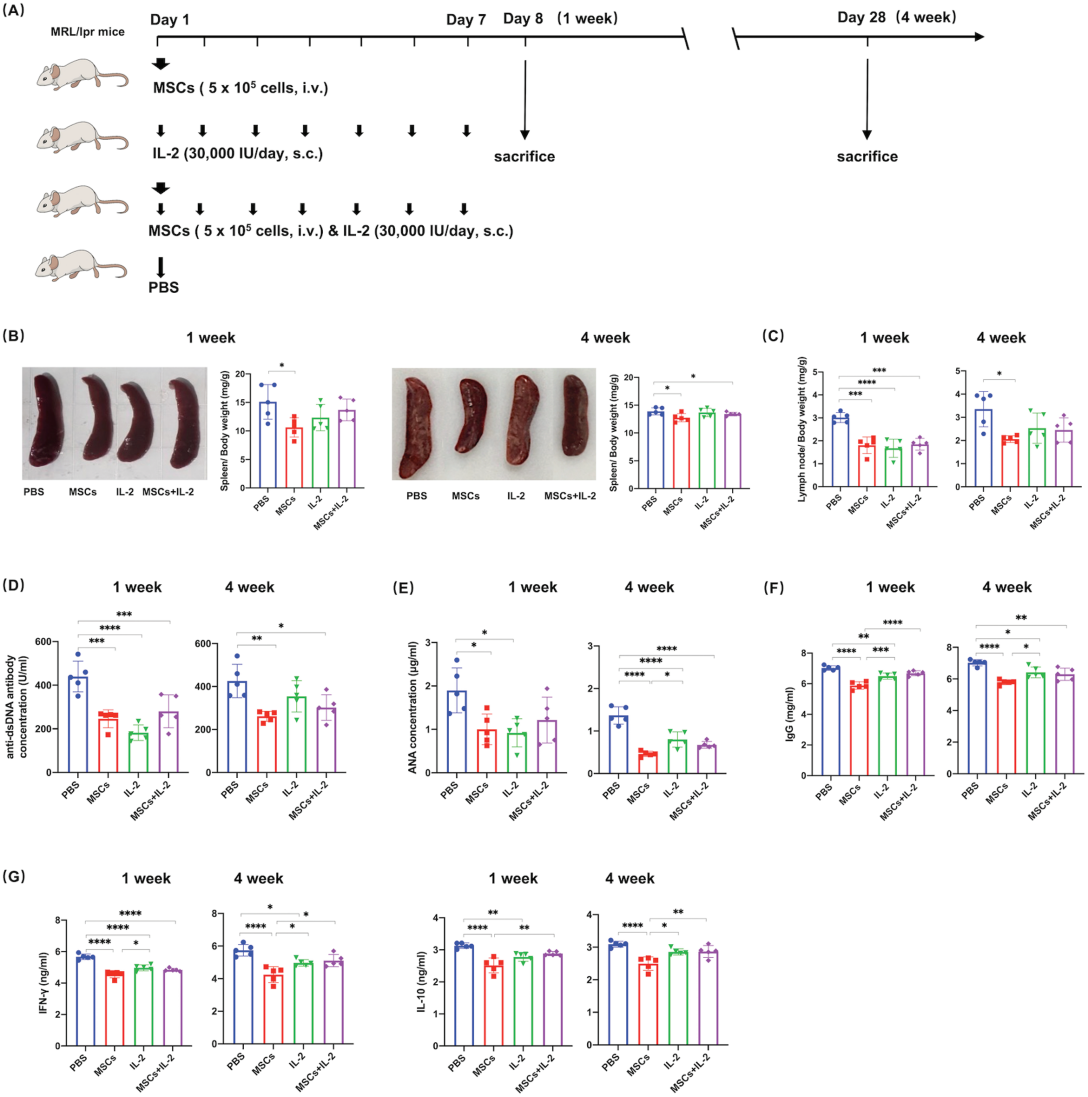
UC-MSCs or low-dose IL-2 treatment alleviated lupus diseases in MRL/lpr mice. (**A**) Schematic illustration of experiments. (**B-C**) Spleen/body weight ratio (mg/g) (B) and lymph nodes/body weight ratio (mg/g) (C) were calculated. (**D-G**) Serum concentrations of anti-dsDNA antibody (D), ANA (E), IgG (F), IFN-γ, and IL-10 (G) in MRL/lpr mice were determined by ELISA. All the experiments were repeated 3 times. *n* = 5, per group. *P < .05, **P < .01, ***P < .001, ****P < .0001.

To compare the therapeutic effects of MSCs and low-dose IL-2 on SLE, the lupus-prone mice (MRL/lpr strain) were used. After confirmation of the onset of lupus by monitoring changes in the weekly urine protein, mice were randomly divided into 4 groups and received PBS, allogenic UC-MSCs, IL-2, and the combination of allogenic UC-MSCs and IL-2 simultaneously, shown in Fig. 1A. In the study by He et al., they carefully investigated the therapeutic effects of low-dose IL-2 on SLE and checked the dose effect of IL-2 on T-cell subpopulations.^23^ At the lowest dose, IL-2 had a negligible effect on Treg cell numbers. Since the object of our study is to compare the therapeutic effects of MSCs and low-dose IL-2 on SLE, we chose the dose (30 000 IU/day subcutaneously for 1 week) used by their study.

Mice were sacrificed 1 or 4 weeks later to evaluate the short- and long-term curative effects. Enlarged spleen and lymph nodes, indicating lymphoid hyperplasia, are the result of an overactivated immune system to an undefined antigenic stimulus.^24,25^ Our data showed that UC-MSCs treatment significantly reduced the size of spleens and lymph nodes and kept such inhibition till 4-week after treatment. However, low-dose IL-2 only had a limited restriction on the growth of lupus lymphoid organs, as the size of spleens did not change significantly after treatment. For lymph nodes, they shrank only during the first week after low-dose IL-2 treatment and regained their original sizes 4 weeks later. For UC-MSCs + IL-2 treatment, it showed comparable therapeutic efficacy to UC-MSCs alone (Fig. 1B, 1C). MRL/lpr mice possess hypergammaglobulinemia and increased titers of anti-nuclear antibodies (ANA) and anti-double stranded DNA antibodies (anti-dsDNA antibodies), which are indicators of systemic humoral autoimmunity.^26^ We found that UC-MSCs strongly suppressed the production of autoantibodies to cellular and nuclear antigen and IgG, while the suppression of IL-2 on anti-dsDNA antibodies was only observed in the first week after treatment and soon disappeared (Fig. 1D–1F). Together, these findings demonstrated that although both UC-MSCs and low-dose IL-2 ameliorated lupus-like symptoms shortly after treatment, the therapeutic effects of UC-MSCs persisted longer. Besides, IL-2 cannot enhance the outcome of UC-MSCs treatment.

During the onset of SLE, inflammatory mediators are produced in large quantities and accelerate the disease’s progression. Therefore, we then looked at how these treatments modulate the production of cytokines closely related to autoimmune diseases (Fig. 1G). Previous studies have shown that interferon-gamma (IFN-γ) was involved in the aggravation of SLE disease activity and kidney damage.^27,28^ Here, we observed that serum IFN-γ decreased soon after all 1-week treatments, with persistent inhibition only seen in the UC-MSCs group. Dysregulation of interleukin-10 (IL-10) is associated with enhanced immunopathology in SLE.^29^ In the present study, all treatment groups showed a significant reduction in serum IL-10. These results indicated that compared to IL-2, UC-MSCs had stronger immunosuppressive functions in MRL/lpr mice.

### UC-MSCs Worked Better in Improving Renal Functions

**Figure 2.**
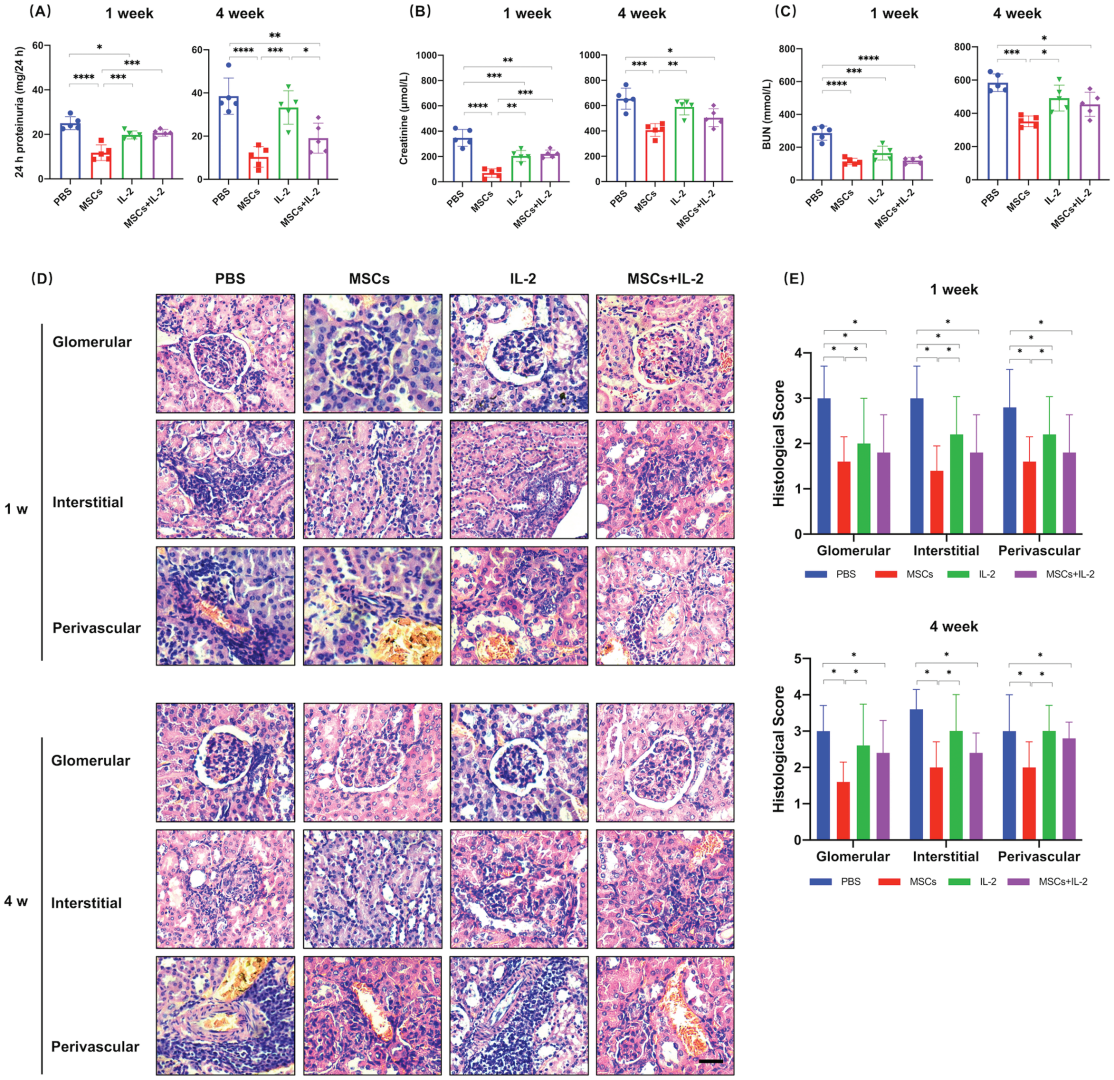
UC-MSCs treatment provided long-term alleviation of renal damage. (**A-C**) 24 h proteinuria (A), serum creatinine (B), and BUN (C) were measured in the respective groups at 1 or 4 weeks after treatment. The level of 24 h proteinuria was expressed as micrograms of protein in 24-h urine. (**D-E**) Glomerular pathology quantified as glomerular, interstitial, and perivascular scores of mice receiving different treatments were determined. (D) Representative images of H&E-stained kidneys. Scale bar, 50 μm. (E) Histological scores of renal lesions were calculated by the severity of glomerulonephritis, interstitial nephritis, and vessels, graded on a 1-4 scale. All the experiments were repeated 3 times. *n* = 5, per group. *P < .05, **P < .01, ***P < .001, ****P < .0001.

**Figure 3.**
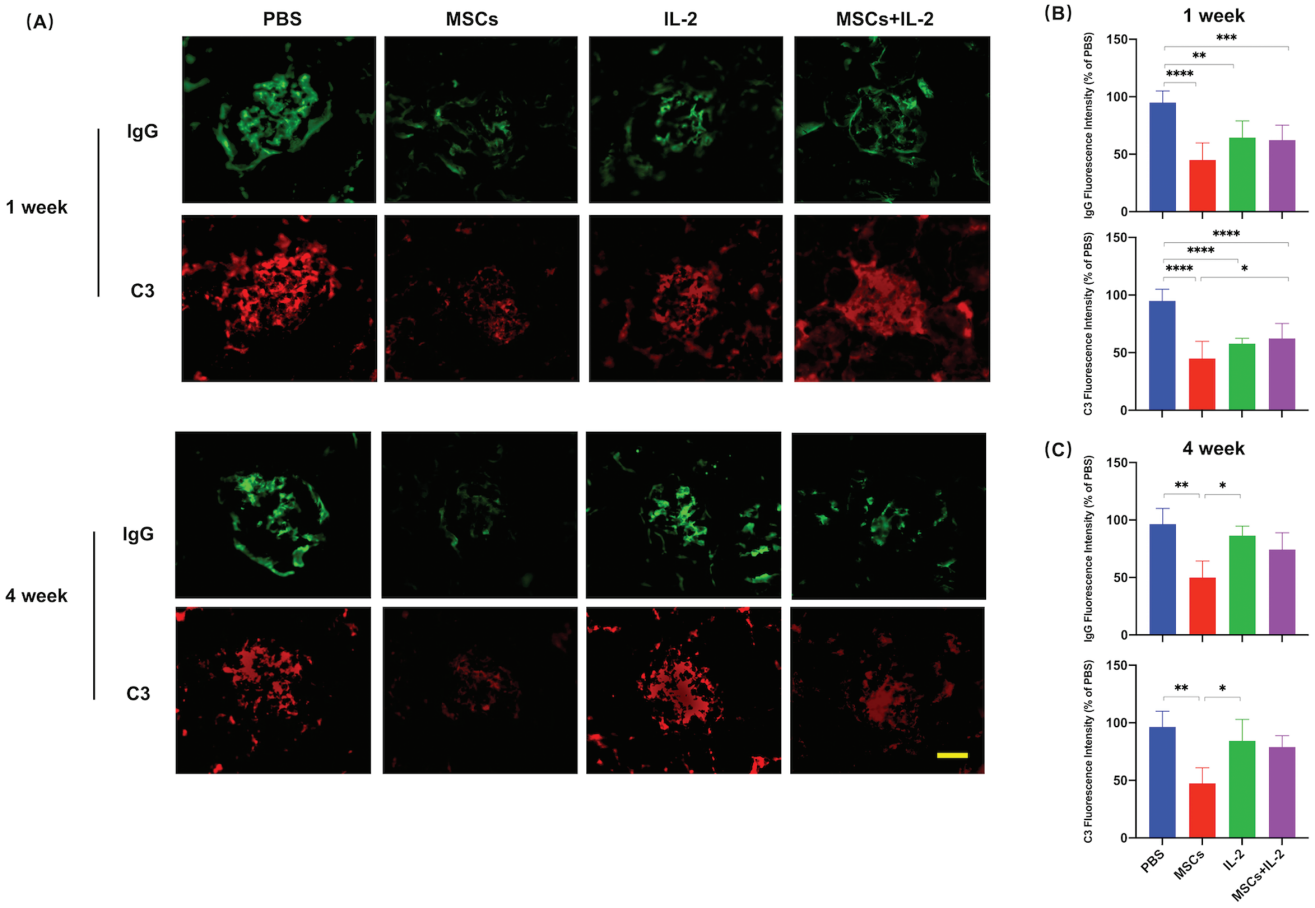
UC-MSCs treatment maintained long-term inhibition of immune complexes deposition. Kidney damage was assessed by evaluation for glomerular immunoglobulin deposition, as well as immunofluorescence intensity. (**A**) Representative kidney immunofluorescence staining of IgG (green) and C3 (red) deposition in the respective groups. Scale bar, 30 μm. (**B-C**) Quantification of IgG (B) and C3 (C) immunofluorescence intensity was normalized to PBS group after analyzed by ImageJ. All the experiments were repeated 3 times. *n* = 5, per group. *P < .05, **P < .01, ***P < .001, ****P < .0001.

Lupus nephritis is a major manifestation of SLE and causes nephrotic syndrome or chronic kidney disease, leading to end-stage renal failure.^30^ Therefore, we evaluated the lupus renal lesions after treatment. Urinalysis showed that UC-MSCs and low-dose IL-2 treatments instantly reduced the level of urine protein in mice. Proteinuria levels decreased more in the UC-MSCs-treated group and were maintained over time, but the IL-2 group returned to the pre-treatment level 4 weeks later (Fig. 2A). UC-MSCs and UC-MSCs + IL-2 treatment groups, which we called UC-MSCs-related treatment groups in the following sections, significantly slowed down the increase of serum creatinine and BUN and alleviated disease exacerbation and lupus nephritis. However, downregulation of serum creatinine and BUN by IL-2 was only observed in the first week after treatment, suggesting that UC-MSCs were a better choice for treating lupus nephritis (Fig. 2B–2C). H&E staining of kidney sections revealed that glomerulonephritis, interstitial nephritis, and infiltration of lymphocytes surrounding blood vessels of all groups were ameliorated at the early time after treatment compared to extensive glomerulonephritis shown in the control groups, and UC-MSCs still provided the longest protection to the kidney (Fig. 2D, 2E). Assessment of glomerular immune complex deposition showed that treatments involved UC-MSCs restricted glomerular IgG and C3 deposition for a long time and were better than low-dose IL-2 (Fig. 3A–3C). These data revealed that although UC-MSCs, low-dose IL-2, and UC-MSCs + IL-2 all improved renal function, only UC-MSCs obtained a sustained repairing effect on the tissue damage of the lupus kidney.

### Influences of UC-MSCs and Low-Dose IL-2 Treatment on T-Cell Responses in Lupus Mice

**Figure 4.**
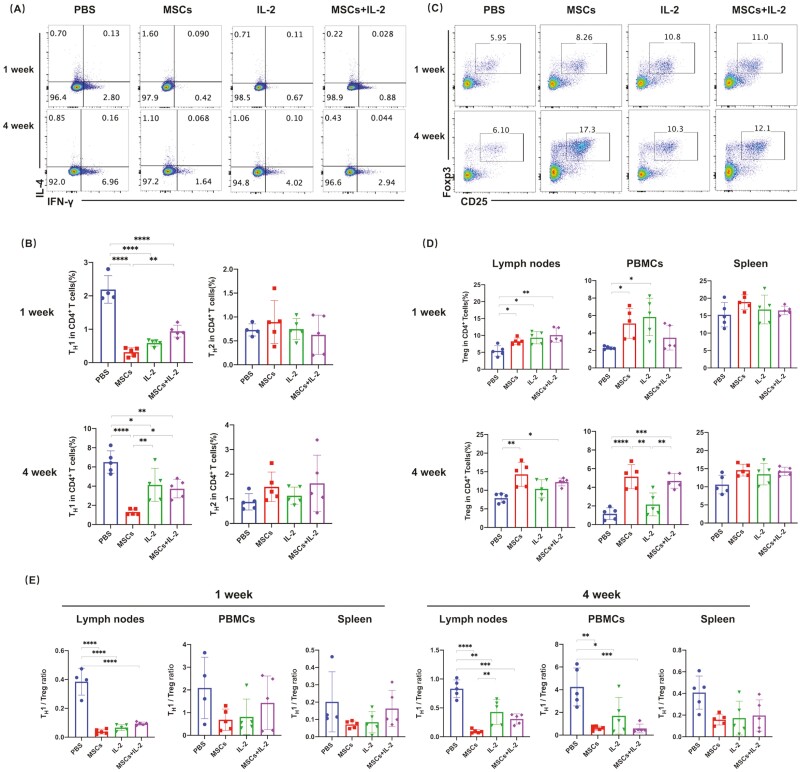
UC-MSCs regulated T-cell subtypes. (**A-B**) Flow cytometric analysis of T_H_1 and T_H_2 cells in the lymph nodes of MRL/lpr mice in the respective groups. (A) Representative staining profiles of T_H_1 and T_H_2 cells and (B) percentages of T_H_1 and T_H_2 cells were shown. (**C-D**) Flow cytometric analysis of Tregs in the spleens, PBMCs, and lymph nodes of MRL/lpr mice in the respective groups. (C) Representative staining profiles of Tregs in the lymph nodes and (D) percentages of Tregs in the lymph nodes, PBMCs, and spleens were shown. (**E**) The ratio of T_H_1/Treg in the lymph nodes, PBMCs, and spleens was shown. PBS group in 1-week treatment (A-B, E), *n* = 4. Other groups, *n* = 5. All the experiments were repeated 3 times. *P < .05, **P < .01, ***P < .001, ****P < .0001. w, week.

The imbalance of T helper cell subsets (T_H_1, T_H_2, and T_H_17) and Tregs are suggested to contribute to the pathogenesis of SLE.^31^ Previous studies demonstrated that T_H_1 cells were the main contributor to the exacerbation of renal lesions in lupus nephritis patients.^32^ UC-MSCs were reported to mainly exert inhibitory effects on the differentiation and effector functions of T_H_1 cells.^33^ Therefore, we first evaluated Th responses in spleens, lymph nodes, and PBMCs of MRL/lpr mice. Our results demonstrated that UC-MSCs-related treatments led to a stronger reduction in the proportion of T_H_1 cells than IL-2 treatment. On the other hand, only a few T_H_2 and T_H_17 cells were detected in lupus mice and were not changed significantly after all the treatments (Fig. 4A, 4B; Supplementary S1, S2). In general, these data illustrated that UC-MSCs had a persistent suppressive impact on T_H_1 responses.

We then explored the influences of UC-MSCs and IL-2 on Treg cell development. Shortly after the treatment, both UC-MSCs and low-dose IL-2 increased the percentages of Tregs in spleens, lymph nodes, and PBMCs. However, for the long-term therapeutic effects, only UC-MSCs-related treatments led to a persistent increase in Tregs. The upregulation of Tregs by IL-2 soon disappeared after stopping the usage of the drug (Fig. 4C–4D). Besides the percentages of Tregs, upregulation of FOXP3 by MSCs has also been observed and correlated with their immunosuppressive effects. However, according to our flow cytometry data, there was no apparent elevation in the mean fluorescence intensity (MFI) of FOXP3 (Supplementary Fig. S9). Thus, the therapeutic effects of MSCs should be attributed to the increased frequencies of Treg cells.

As the ratio is thought to better reflect the T_H_1/Treg balance in the different tissues, we also checked it and found that all the treatments could downregulate the T_H_1/Treg ratio in lymph nodes and the downregulation lasted for 4 weeks. Although the regulation in peripheral blood and spleen was not statistically significant, the imbalance of Th1/Treg was reversed by treatments. There was no obvious difference between treatments (Fig. 4E). Except for Tregs, other regulatory Treg-cell subsets (nTreg, iTreg, and CD8^+^ Treg) and regulatory innate lymphoid cells (ILCreg) have been proven to play important roles in maintaining homeostasis. Therefore, we also examined the influences of the treatments on these cells and found no difference before and after treatment (Supplementary Fig. S4).

### IL-2 Was Involved in the Upregulation of Tregs by UC-MSCs

**Figure 5.**
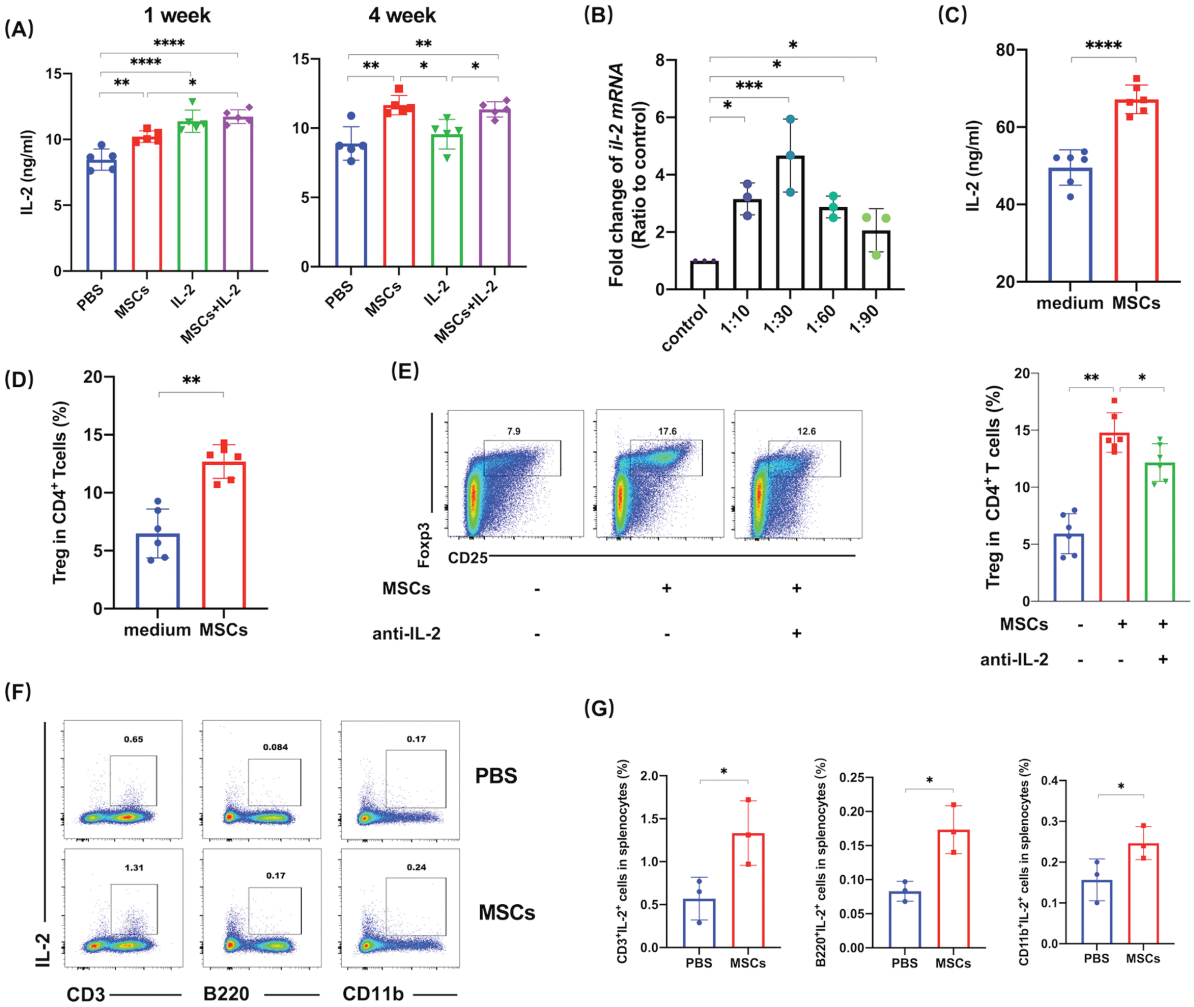
IL-2 was involved in MSCs-induced Tregs. (**A**) Serum IL-2 of MRL/lpr mice in the respective groups was determined by ELISA. (**B**) Splenocytes of C57BL/6 mice were co-cultured with MSCs in vitro at different ratios and 12 h later, fold change of *il-2* mRNA was determined by qRT-PCR. (**C-D**) Splenocytes of C57BL/6 mice were co-cultured with MSCs at 1:30 (as MSCs: splenocytes) and 24 h later, (C) concentrations of IL-2 in culture supernatants were measured by ELISA and (D) percentages of Tregs were determined by FACS. (**E**) Splenocytes of C57BL/6 mice were co-cultured with MSCs in the presence of IL-2 neutralizing antibodies or not, then were harvested 24 h later. Percentages of Tregs were determined. (**F-G**) Frequencies of CD3^+^IL-2^+^, B220^+^IL-2^+^, and CD11b^+^IL-2^+^cells in splenocytes from mice treated with MSCs and PBS for 4 weeks. All the experiments were repeated 3 times. *n* = 3-6, per group. *P < .05, **P < .01, ***P < .001, ****P < .0001.

In many studies, soluble factors such as TGF-β, IGFBP-4, CTLA4, and PGE2 have been identified to play important roles in the upregulation of Tregs by UC-MSCs.^13-16^ IL-2 is also one of the molecules regulating Tregs, but whether UC-MSCs could regulate IL-2 and then promote the generation of Tregs remains unknown. In this way, we measured and compared the serum level of IL-2 in mice after treatment. UC-MSCs were found to cause a long-lasting rise of IL-2. Low-dose IL-2 can only maintain the elevation of serum IL-2 for a short period. When the drug was withdrawn, serum IL-2 soon dropped to the original level, indicating that continuous usage is required to maintain blood concentration (Fig. 5A). This finding is consistent with the previous clinical trials of low-dose IL-2 in SLE patients.^34,35^ Then, we tested the hypothesis that IL-2 was involved in the UC-MSCs-induced increase of Tregs. We found that co-cultured with UC-MSCs in vitro promoted the expression of *il-2* by mouse splenocytes (Fig. 5B). In line with the gene expression data, IL-2 in the culture supernatant significantly increased (Fig. 5C). Importantly, the proportion of Tregs in the splenocytes increased as well (Fig. 5D). To further validate that UC-MSCs-induced IL-2 is indispensable for Treg-cell escalation, we added IL-2 neutralizing antibodies into the co-culture system and found that the upregulation of Tregs caused by UC-MSCs diminished but not totally vanished (Fig. 5E), suggesting that IL-2 was partly involved in the UC-MSCs-induced rise of Tregs. Then, we determined the cell subsets that were responsible for the increased production of IL-2. The data showed that it was CD3^+^ T cells that produced IL-2 rather than other cell subsets in the spleen (Fig. 5F–5G). TGF-β has previously been reported to be involved in UC-MSCs upregulation of Tregs.^36^ Therefore, we also measured serum TGF-β in the mice and found an increase compared to the control group (Supplementary Fig. S3), suggesting that TGF-β may act synergistically with IL-2 and other soluble factors or immune cells to maintain the long-term upregulation of Tregs by UC-MSCs.

**Figure 6.**
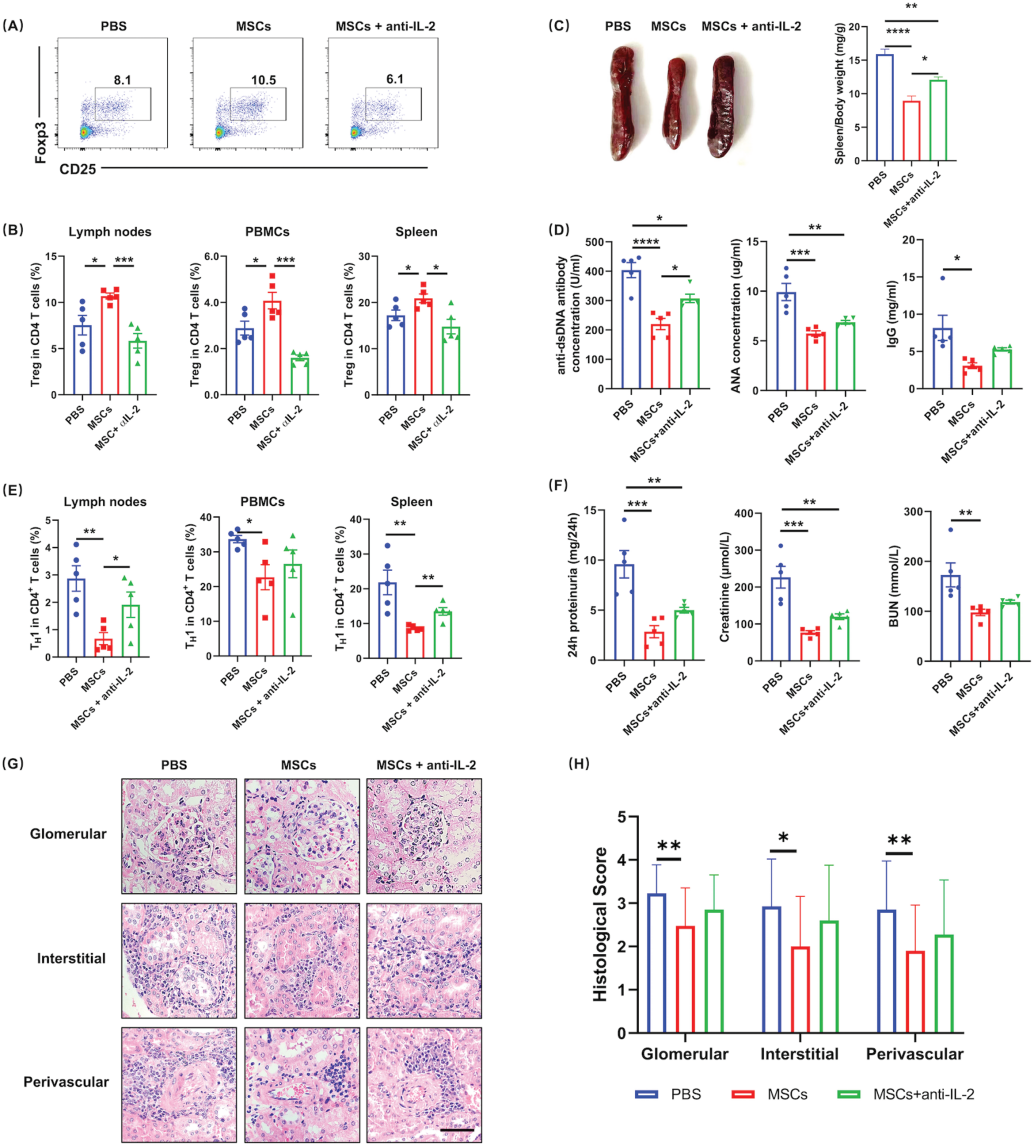
Neutralization of IL-2 significantly impaired the therapeutic effects of MSCs on SLE. After receiving MSCs, MRL/lpr mice were treated with IL-2 neutralizing antibodies or istotype IgG. Mice that received PBS only were set as control. One week later, lymph nodes, PBMCs, and spleens were collected. (**A-B**) Percentages of Tregs were determined by FACS and representative staining profiles of lymph nodes were shown. (**C**) Spleens were weighed and spleen/body weight ratio (mg/g) was calculated. (**D**) Serum anti-dsDNA antibody, ANA, and IgG were determined by ELISA. (**E**) T_H_1 in the lymph nodes, PBMCs, and spleens were determined by FACS. (**F**) 24 h proteinuria, serum creatinine, and BUN were measured. The level of 24 h proteinuria was expressed as micrograms of protein in 24-h urine. (**G**) Representative images of H&E-stained kidneys. Scale bar, 100 μm. (**H**) Glomerular pathology quantified as glomerular, interstitial, and perivascular scores of mice receiving different treatments were determined. All the experiments were repeated 3 times. *n* = 5, per group. *P < .05, **P < .01, ***P < .001, ****P < .0001.

To further confirm the role of IL-2 in the regulation of MSCs on Tregs in vivo, we treated MRL/lpr mice with IL-2 neutralizing antibodies soon after the intravenous transfer of MSCs. Consistent with the in vitro observation, we found that IL-2 deprivation significantly abolished the increase of Tregs (Fig. 6A, 6B) and weakened the therapeutic effects of MSCs. Compared with MSC-treated mice, those who received both MSC and anti-IL-2 antibodies had higher spleen/body weight ratio, serum autoantibodies (anti-dsDNA antibodies and ANA), and T_H_1 responses (Fig. 6C–6E). Moreover, renal function restoration by MSCs also declined when IL-2 was neutralized (Fig. 6F–6H). Together, our results provided evidence that UC-MSCs led to prolonged production of IL-2, which subsequently supported Treg-cell upregulation.

### An Increase of IL-2 Positively Correlated With the Disease Remission of Lupus Patients After Receiving UC-MSCs

**Figure 7.**
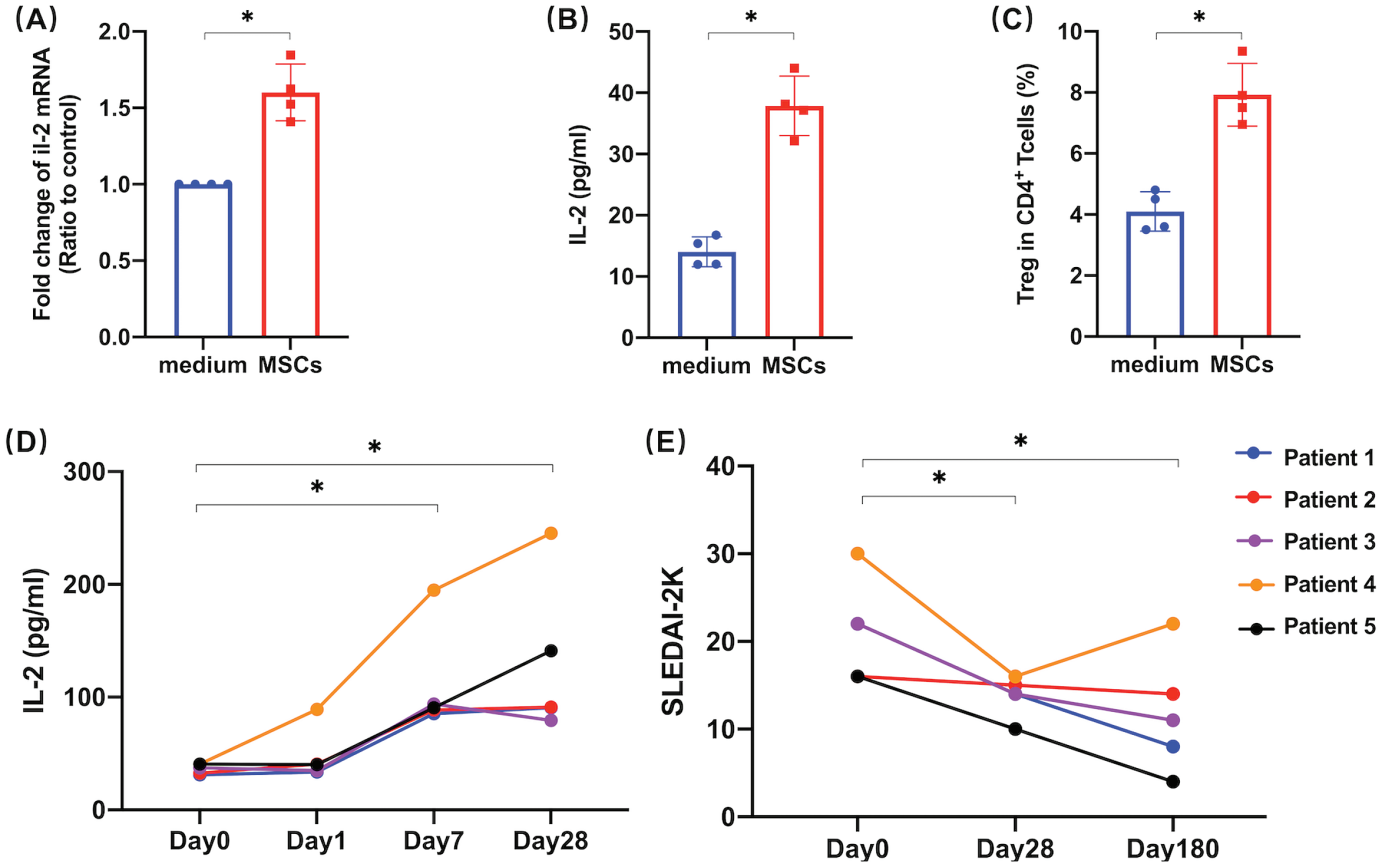
Higher IL-2 maintaining positively correlated with therapeutic efficacy of UC-MSCs treatment in lupus nephritis patients. (**A-C**) PBMCs were isolated from 4 SLE patients, then were co-cultured with MSCs at the ratio of 1:30 (as MSCs: PBMCs) for 12 h (A) and 24 h (B-C), respectively. (A) PBMCs were then harvested for further examination of their *il-2 mRNA* expression by qRT-PCR. (B) IL-2 concentration in the culture supernatant was measured by ELISA. (C) Frequencies of Tregs (CD4^+^CD25^+^FoxP3^+^cells) for the indicated subsets were determined. (**D-E**) Serum IL-2 concentration (D) and SLEDAI-2K scores (E) of 5 lupus nephritis patients treated with MSCs were collected. *P < .05, **P < .01, ***P < .001, ****P < .0001.

Since all the data mentioned above were obtained from the murine study, we wanted to know whether similar results were obtained in the SLE patients treated with MSC-based therapy. Therefore, we enrolled 4 patients and isolated their PBMCs, and co-cultured the cells with UC-MSCs. The data showed that UC-MSCs increased IL-2 in both gene and protein levels (Fig. 7A–7B). Flow cytometry data also confirmed the increase of Tregs in PBMCs after co-culturing with UC-MSCs (Fig. 7C). To determine the relationship between the increase of IL-2 and the therapeutic effects of UC-MSCs in vivo, 5 refractory lupus patients were recruited. Patients were examined before the UC-MSCs treatment and during 3 follow-up visits on 1 day, 1, and 4 weeks after that. We found a significant increase in serum IL-2 sustained for 1 month, meanwhile, SLEDAI-2K scores also decreased. At 6 months post-UC-MSCs-treatment, the SLEDAI-2K score slightly increased compared with its previous visit, though it still was significantly lower than the baseline (Fig. 7D–7E). Together, the present findings implied that UC-MSCs increased Tregs by promoting IL-2 production and alleviated disease activity for a long time.

## Discussion

SLE is a chronic multisystem autoimmune disease and its empirical treatments mainly rely on the use of glucocorticoids and immunosuppressants.^2^ However, 5%-20% of SLE patients are refractory to this treatment.^37^ Novel biological therapies emerged in recent years and among them, both MSCs and low-dose IL-2 treatment show great potential.^3,21^ The mechanisms for MSCs and IL-2 in treating SLE are under extensive study but still not fully understood. In this study, we provided in vivo data by performing a head-to-head comparison of UC-MSCs and low-dose IL-2 treatment in MRL/lpr mice. We also tested if there are additive effects by combining UC-MSCs and IL-2 treatment. Lastly, we provided in vitro, in vivo, and clinical evidence suggesting the therapeutic efficacy of UC-MSCs is directly correlated with increased serum IL-2 levels.

The therapeutic effects of low-dose IL-2 through targeting Treg in treating SLE have been reported in several preclinical and clinical studies.^38,39^ However, due to its short half-life and rapid clearance by kidney,^40,41^ one disadvantage of low-dose IL-2 therapy is that it requires continuous administration. Consistent with this, we also found that IL-2 treatment resulted in increased IL-2 concentration 1 week after treatment, but the level returned to the control level 4 weeks after treatment. Also, in the IL-2-treated group, the Treg percentage increased in 1 week and decreased in 4 weeks. In all, continuous administration is required for the maintenance of IL-2 treatment. In contrast, a single intravenous injection of UC-MSCs provided longer therapeutic effects compared with low-dose IL-2. Four weeks after treatment, only groups treated with MSCs displayed remarkably prolonged effects on mitigating lupus-like autoimmunity symptoms. This is consistent with our group’s previous reports, showing single MSCs treatment provides long-term therapeutic efficacy in multiple clinical trials.^5-7^

Notably, conventional treatments may not exhibit quick therapeutic effects on kidney pathology and autoantibody production including ANA which in humans typically does not decrease with effective therapy. However, for MSCs, we found that the treatment resulted in a significant reduction of ANA levels and an improvement in renal function within a week of treatment. This was consistent with what we observed in a clinical MSC trial that enrolled 15 patients with refractory SLE.^42^ The data was very impressive, showing that MSCs have great tissue-repairing and antibody-inhibitory functions. Moreover, these function lasts for quite a long time. Ruan et al. showed in a murine lupus model that in addition to 24-h urine protein levels, anti-nuclear, anti-histone, and anti-dsDNA antibody levels all decreased at 4 weeks after UC-MSC transplantation.^43^ In a clinical study, Deng et al. also reported a reduction in ANA titers and renal improvement in SLE patients 6 months after UC-MSC infusion.^44^ Although the underlying mechanism of the antibody-inhibitory effects of MSCs remained largely unknown, our previous study on olfactory 1/early B cell factor-associated zinc-finger protein (OAZ) shed light.^45^ We found that the knockdown of OAZ expression could restore the impaired MSC function in regulating B cells and lead to a decrease in ANA levels. Therefore, MSC treatment may be a promising modality for reducing autoantibody production in SLE patients. Moreover, the reduction of ANA levels may serve as a potential manifestation of the therapeutic efficacy of MSCs in SLE.

The mechanism of UC-MSCs in treating SLE is not fully understood and for the first time, we provided evidence suggesting that MSCs treatment might relieve SLE manifestations through up-regulating IL-2, which plays an important role in promoting the expansion of Tregs.^22,41,46^ However, to our best knowledge, no direct evidence show that IL-2 is involved in the upregulation of Tregs by UC-MSCs. Here, we showed that UC-MSCs treatment increased both serum IL-2 levels and the percentage of Tregs both in vitro and in vivo and the increase of IL-2 resulting from UC-MSCs treatment lasted for at least 4 weeks. In SLE patients receiving UC-MSCs, we also found that elevated IL-2 was associated with remission of the disease. In all, these observations indicated that UC-MSCs promote the progress of Tregs by elevating IL-2 production.

The mechanism of UC-MSCs regulation of IL-2 and Treg remains unclear and is worth further investigation. Based on previous studies, UC-MSCs might secrete soluble factors such as fibroblast growth factor-1 (FGF-1), and IL-33, or promote the production of IL-12 by macrophages, monocytes, and dendritic cells to promote IL-2 production by T cells.^47-51^ However, our results showed that deprivation of IL-2 did not totally counteract the increase of Tregs by UC-MSCs, suggesting that other factors are also indispensable. Indeed, we also detected a slight increase of TGF-β compared to the control group, which is one of the strongest factors for promoting Tregs^52^ after UC-MSCs treatment. This suggests the regulation of Tregs by UC-MSCs might involve multiple factors besides IL-2.

In this study, we also tested if there is an additive effect for MSCs treatment in combination with low-dose IL-2 treatment and it turned out that they were not better than either UC-MSCs or IL-2 alone, suggesting IL-2 level is critical for the therapeutic effects for both UC-MSCs and IL-2 treatments. One week after treatment, the UC-MSCs + IL-2 group showed higher IL-2 levels compared with either UC-MSCs or IL-2 alone. But the higher level of serum IL-2 did not translate to better alleviation of diseases. One possibility is that serum IL-2 levels in the combination group might already exceed an optimal range of serum IL-2 and turn out to be detrimental. In fact, it has been shown that a high-dose IL-2 would result in a cascade of cytokines released at supraphysiologic levels from IL-2-activated cells leading to a well-described capillary leak syndrome and eventual end-organ dysfunction.^53^ Herein, we indeed found that cells from the UC-MSCs + IL-2 group exhibited higher activation levels, as CD8^+^ T and NK cells expressed higher CD69 and produced much more cytotoxic molecules, perforin (Supplementary Fig. S7, S8). According to the study by Galleu et al., these activated cytotoxic cells may induce the apoptosis of MSCs via bystander effect.^54^ However, we and others have shown that the renal protective effects of MSCs are linked to the capacity to migrate to the site of renal damage and to release extracellular vesicles and pro-survival, anti-inflammatory, and immunomodulatory factors locally.^55,56^ Thus, the less effectiveness of MSCs + IL-2 in kidney damage repairing and lupus symptom alleviation may be due to the quick loss of MSCs. At 4 weeks, serum IL-2 level was lowest in the IL-2 treated group alone. This is consistent with the rapid clearance of IL-2 as mentioned above since low-dose IL-2 was administered 4 weeks ago. A similar level of serum IL-2 was detected in both the UC-MSCs + IL-2 group and the UC-MSCs group alone, consistent with the observation that the combination therapy showed similar effects compared with UC-MSCs treatment alone. Taken together, all these data suggest that both low-dose IL-2 and MSCs treatment showed similar efficacy to induce rapid expansion of Tregs and suited for the treatment during acute disease exacerbations. While for the treatment of chronic diseases, including autoimmune diseases that need long-term sustaining of Tregs and inhibition of inflammation, MSCs treatment is preferable.

In summary, by systemically comparing the therapeutic effects of MSCs treatment and low-dose IL-2, we showed that both IL-2 and UC-MSCs promote the increase of Tregs. A single dose of UC-MSCs treatment provided a long-lasting effect in reducing lupus-like autoimmunity compared with low-dose IL-2 treatment. UC-MSCs treatment resulted in increased serum IL-2 levels in vitro, in vivo, and in patients. Optimal serum IL-2 level was critical for the therapeutic effects of both low-dose IL-2 and UC-MSCs treatment. A randomized controlled clinical trial would be needed to further compare the 2 treatments.

## Supplementary Material

szad032_suppl_Supplementary_Materials

## Data Availability

All data relevant to the study are included in the article.

## References

[1] Fava A , Petri M. Systemic lupus erythematosus: diagnosis and clinical management. J Autoimmun. 2019;96:1-13. 10.1016/j.jaut.2018.11.001 30448290 PMC6310637

[2] Tsokos GC. Systemic lupus erythematosus. N Engl J Med. 2011;365(22):2110-2121. 10.1056/NEJMra1100359 22129255

[3] Merimi M , Lewalle P, Meuleman N, et al. Mesenchymal stem/stromal cell therapeutic features: the bridge between the bench and the clinic. J Clin Med. 2021;10(5):905. 33668878 10.3390/jcm10050905 PMC7956428

[4] Wang Y , Tian M, Wang F, et al. Understanding the immunological mechanisms of mesenchymal stem cells in allogeneic transplantation: from the aspect of major histocompatibility complex class I. Stem Cells Dev. 2019;28(17):1141-1150. 10.1089/scd.2018.0256 31215341

[5] Wang D , Li J, Zhang Y, et al. Umbilical cord mesenchymal stem cell transplantation in active and refractory systemic lupus erythematosus: a multicenter clinical study. Arthritis Res Ther. 2014;16(2):R79. 10.1186/ar4520 24661633 PMC4060570

[6] Sun L , Wang D, Liang J, et al. Umbilical cord mesenchymal stem cell transplantation in severe and refractory systemic lupus erythematosus. Arthritis Rheum. 2010;62(8):2467-2475. 10.1002/art.27548 20506343

[7] Wang D , Zhang H, Liang J, et al. Allogeneic mesenchymal stem cell transplantation in severe and refractory systemic lupus erythematosus: 4 years of experience. Cell Transplant. 2013;22(12):2267-2277. 10.3727/096368911X582769c 24388428 PMC11849135

[8] Zhou T , Li HY, Liao C, Lin W, Lin S. Clinical efficacy and safety of mesenchymal stem cells for systemic lupus erythematosus. Stem Cells Int. 2020;2020:6518508. 10.1155/2020/6518508 32322279 PMC7157802

[9] Baiguera S , Jungebluth P, Mazzanti B, Macchiarini P. Mesenchymal stromal cells for tissue-engineered tissue and organ replacements. Transpl Int. 2012;25(4):369-382. 10.1111/j.1432-2277.2011.01426.x 22248229

[10] Ohl K , Tenbrock K. Regulatory T cells in systemic lupus erythematosus. Eur J Immunol. 2015;45(2):344-355. 10.1002/eji.201344280 25378177

[11] Uccelli A , de Rosbo NK. The immunomodulatory function of mesenchymal stem cells: mode of action and pathways. Ann N Y Acad Sci. 2015;1351:114-126. 10.1111/nyas.12815 26152292

[12] Doyle EC , Wragg NM, Wilson SL. Intraarticular injection of bone marrow-derived mesenchymal stem cells enhances regeneration in knee osteoarthritis. Knee Surg Sports Traumatol Arthrosc. 2020;28(12):3827-3842. 10.1007/s00167-020-05859-z 32006075 PMC7669782

[13] González MA , Gonzalez-Rey E, Rico L, Büscher D, Delgado M. Adipose-derived mesenchymal stem cells alleviate experimental colitis by inhibiting inflammatory and autoimmune responses. Gastroenterology. 2009;136(3):978-989. 10.1053/j.gastro.2008.11.041 19135996

[14] Court AC , Le-Gatt A, Luz-Crawford P, et al. Mitochondrial transfer from mscs to T cells induces treg differentiation and restricts inflammatory response. EMBO Rep. 2020;21(2):e48052. 10.15252/embr.201948052 31984629 PMC7001501

[15] Miyagawa I , Nakayamada S, Nakano K, et al. Induction of regulatory T cells and its regulation with insulin-like growth factor/insulin-like growth factor binding protein-4 by human mesenchymal stem cells. The Journal of Immunology. 2017;199(5):1616-1625. 10.4049/jimmunol.1600230 28724578

[16] Yang F , Ni B, Liu Q, et al. Human umbilical cord-derived mesenchymal stem cells ameliorate experimental colitis by normalizing the gut microbiota. Stem Cell Res Ther. 2022;13(1):475. 10.1186/s13287-022-03118-1 36104756 PMC9476645

[17] Mizui M , Tsokos GC. Low-dose Il-2 in the treatment of lupus. Curr Rheumatol Rep. 2016;18(11):68. 27734211 10.1007/s11926-016-0617-5

[18] Koreth J , Matsuoka K, Kim HT, et al. Interleukin-2 and regulatory T cells in graft-versus-host disease. N Engl J Med. 2011;365(22):2055-2066. 10.1056/NEJMoa1108188 22129252 PMC3727432

[19] Lieberman LA , Tsokos GC. The Il-2 defect in systemic lupus erythematosus disease has an expansive effect on host immunity. J Biomed Biotechnol. 2010;2010:740619. 10.1155/2010/740619 20625413 PMC2896881

[20] Tsokos GC , Lo MS, Costa Reis P, Sullivan KE. New insights into the immunopathogenesis of systemic lupus erythematosus. Nat Rev Rheumatol. 2016;12(12):716-730. 10.1038/nrrheum.2016.186 27872476

[21] He J , Zhang R, Shao M, et al. Efficacy and safety of low-dose Il-2 in the treatment of systemic lupus erythematosus: a randomised, double-blind, placebo-controlled trial. Ann Rheum Dis. 2020;79(1):141-149. 10.1136/annrheumdis-2019-215396 31537547 PMC6937406

[22] Zhao Z , Zhang X, Su L, et al. Fine tuning subsets of Cd4(+) T cells by low-dosage of Il-2 and a new therapeutic strategy for autoimmune diseases. Int Immunopharmacol. 2018;56:269-276. 10.1016/j.intimp.2018.01.042 29414661

[23] He J , Zhang X, Wei Y, et al. Low-dose interleukin-2 treatment selectively modulates Cd4(+) T cell subsets in patients with systemic lupus erythematosus. Nat Med. 2016;22(9):991-993. 10.1038/nm.4148 27500725

[24] de Porto AP , Lammers AJ, Bennink RJ, et al. Assessment of splenic function. Eur J Clin Microbiol Infect Dis. 2010;29(12):1465-1473. 10.1007/s10096-010-1049-1 20853172 PMC2995208

[25] Wang Y , Xu X, Ran M, et al. The enlargement of abdominal lymph nodes is a characteristic of autoimmune liver disease. Mediators Inflamm. 2020;2020:3631625. 10.1155/2020/3631625 32273828 PMC7115048

[26] Karnopp TE , Chapacais GF, Freitas EC, Monticielo OA. Lupus animal models and neuropsychiatric implications. Clin Rheumatol. 2021;40(7):2535-2545. 10.1007/s10067-020-05493-7 33155159

[27] Chasset F , Arnaud L. Targeting interferons and their pathways in systemic lupus erythematosus. Autoimmun Rev. 2018;17(1):44-52. 10.1016/j.autrev.2017.11.009 29108825

[28] Barrat FJ , Crow MK, Ivashkiv LB. Interferon target-gene expression and epigenomic signatures in health and disease. Nat Immunol. 2019;20(12):1574-1583. 10.1038/s41590-019-0466-2 31745335 PMC7024546

[29] Iyer SS , Cheng G. Role of interleukin 10 transcriptional regulation in inflammation and autoimmune disease. Crit Rev Immunol. 2012;32(1):23-63. 10.1615/critrevimmunol.v32.i1.30 22428854 PMC3410706

[30] Tektonidou MG , Dasgupta A, Ward MM. Risk of end-stage renal disease in patients with lupus nephritis, 1971-2015: a systematic review and Bayesian meta-analysis. Arthritis Rheumatol. 2016;68(6):1432-1441. 26815601 10.1002/art.39594 PMC5071782

[31] Robak E , Niewiadomska H, Robak T, et al. Lymphocyctes Tgammadelta in clinically normal skin and peripheral blood of patients with systemic lupus erythematosus and their correlation with disease activity. Mediators Inflamm. 2001;10(4):179-189. 10.1080/09629350124724 11577994 PMC1781712

[32] Morimoto S , Tokano Y, Kaneko H, et al. The increased interleukin-13 in patients with systemic lupus erythematosus: relations to other Th1-, Th2-related cytokines and clinical findings. Autoimmunity. 2001;34(1):19-25. 10.3109/08916930108994122 11681489

[33] Duffy MM , Ritter T, Ceredig R, Griffin MD. Mesenchymal stem cell effects on T-cell effector pathways. Stem Cell Res Ther. 2011;2(4):34. 10.1186/scrt75 21861858 PMC3219065

[34] von Spee-Mayer C , Siegert E, Abdirama D, et al. Low-dose interleukin-2 selectively corrects regulatory T cell defects in patients with systemic lupus erythematosus. Ann Rheum Dis. 2016;75(7):1407-1415. 10.1136/annrheumdis-2015-207776 26324847

[35] Humrich JY , Riemekasten G. Low-dose interleukin-2 therapy for the treatment of systemic lupus erythematosus. Curr Opin Rheumatol. 2019;31(2):208-212. 10.1097/BOR.0000000000000575 30562181

[36] Zhang Q , Fu L, Liang Y, et al. Exosomes originating from Mscs stimulated with Tgf-Β and Ifn-Γ promote Treg differentiation. J Cell Physiol. 2018;233(9):6832-6840. 10.1002/jcp.26436 29336475 PMC13482522

[37] Kronbichler A , Brezina B, Gauckler P, et al. Refractory lupus nephritis: when, why and how to treat. Autoimmun Rev. 2019;18(5):510-518. 30844548 10.1016/j.autrev.2019.03.004

[38] Rosenzwajg M , Lorenzon R, Cacoub P, et al. Immunological and clinical effects of low-dose interleukin-2 across 11 autoimmune diseases in a single, open clinical trial. Ann Rheum Dis. 2019;78(2):209-217. 10.1136/annrheumdis-2018-214229 30472651

[39] Peterson LB , Bell CJM, Howlett SK, et al. A long-lived Il-2 mutein that selectively activates and expands regulatory T cells as a therapy for autoimmune disease. J Autoimmun. 2018;95:1-14. 10.1016/j.jaut.2018.10.017 30446251 PMC6284106

[40] Zhang B , Sun J, Wang Y, et al. Site-specific pegylation of interleukin-2 enhances immunosuppression via the sustained activation of regulatory T cells. Nat Biomed Eng. 2021;5(11):1288-1305. 10.1038/s41551-021-00797-8 34580438

[41] Yang JH , Cutler AJ, Ferreira RC, et al. Natural variation in interleukin-2 sensitivity influences regulatory T-cell frequency and function in individuals with long-standing type 1 diabetes. Diabetes. 2015;64(11):3891-3902. 10.2337/db15-0516 26224887 PMC4975524

[42] Liang J , Zhang H, Hua B, et al. Allogenic mesenchymal stem cells transplantation in refractory systemic lupus erythematosus: a pilot clinical study. Ann Rheum Dis. 2010;69(8):1423-1429. 10.1136/ard.2009.123463 20650877

[43] Ruan G-p , Yao X, Yang S-j, et al. Transplanted human umbilical cord mesenchymal stem cells facilitate lesion repair in b6.FAS mice. Journal of Immunology Research. 2014;2014 1530501-1530513. 10.1155/2014/530501 PMC4352485 25759830

[44] Deng D , Zhang P, Guo Y, Lim TO. A randomised double-blind, placebo-controlled trial of allogeneic umbilical cord-derived mesenchymal stem cell for lupus nephritis. Ann Rheum Dis. 2017;76(8):1436-1439. 10.1136/annrheumdis-2017-211073 28478399

[45] Feng X , Che N, Liu Y, et al. Restored immunosuppressive effect of mesenchymal stem cells on B cells after olfactory 1/early B cell factor-associated zinc-finger protein down-regulation in patients with systemic lupus erythematosus. Arthritis Rheumatol 2014;66(12):3413-3423. 10.1002/art.38879 25219468

[46] Taylor EB , Sasser JM, Maeda KJ, Ryan MJ. Expansion of regulatory T cells using low-dose interleukin-2 attenuates hypertension in an experimental model of systemic lupus erythematosus. Am J Physiol Renal Physiol. 2019;317(5):F1274-F1284. 10.1152/ajprenal.00616.2018 30892934 PMC6879936

[47] Matta BM , Lott JM, Mathews LR, et al. Il-33 Is an unconventional alarmin that stimulates Il-2 secretion by dendritic cells to selectively expand Il-33r/St2+ regulatory T cells. J Immunol. 2014;193(8):4010-4020. 10.4049/jimmunol.1400481 25217167 PMC4185240

[48] Rana BMJ , Jou E, Barlow JL, et al. A stromal cell niche sustains Ilc2-mediated type-2 conditioning in adipose tissue. J Exp Med. 2019;216(9):1999-2009. 10.1084/jem.20190689 31248899 PMC6719433

[49] Wu L , Leijten J, van Blitterswijk CA, Karperien M. Fibroblast growth factor-1 is a mesenchymal stromal cell-secreted factor stimulating proliferation of osteoarthritic chondrocytes in co-culture. Stem Cells Dev. 2013;22(17):2356-2367. 10.1089/scd.2013.0118 23557133 PMC3749707

[50] Li L , Hsu HC, Stockard CR, et al. Il-12 inhibits thymic involution by enhancing Il-7- and Il-2-induced thymocyte proliferation. J Immunol. 2004;172(5):2909-2916. 10.4049/jimmunol.172.5.2909 14978093

[51] Bollyky PL , Falk BA, Long SA, et al. Cd44 costimulation promotes Foxp3+ regulatory T cell persistence and function via production of Il-2, Il-10, and Tgf-beta. J Immunol. 2009;183(4):2232-2241. 10.4049/jimmunol.0900191 19635906 PMC3057032

[52] Xu A , Liu Y, Chen W, et al. Tgf-Β-induced regulatory T cells directly suppress B cell responses through a noncytotoxic mechanism. J Immunol. 2016;196(9):3631-3641. 10.4049/jimmunol.1501740 27001954 PMC4868785

[53] Rosenberg SA , Lotze MT, Muul LM, et al. Observations on the systemic administration of autologous lymphokine-activated killer cells and recombinant interleukin-2 to patients with metastatic cancer. N Engl J Med. 1985;313(23):1485-1492. 10.1056/NEJM198512053132327 3903508

[54] Galleu A , Riffo-Vasquez Y, Trento C, et al. Apoptosis in mesenchymal stromal cells induces in vivo recipient-mediated immunomodulation. Sci Transl Med. 2017;9(416):eaam7828. 29141887 10.1126/scitranslmed.aam7828

[55] Geng L , Tang X, Zhou K, et al. Microrna-663 induces immune dysregulation by inhibiting Tgf-Β1 production in bone marrow-derived mesenchymal stem cells in patients with systemic lupus erythematosus. Cell Mol Immunol. 2019;16(3):260-274. 10.1038/cmi.2018.1 30886422 PMC6460486

[56] Perico L , Morigi M, Rota C, et al. Human mesenchymal stromal cells transplanted into mice stimulate renal tubular cells and enhance mitochondrial function. Nat Commun. 2017;8(1):983. 10.1038/s41467-017-00937-2 29042548 PMC5754365

