# Correction to: Allogenic Umbilical Cord-Derived Mesenchymal Stromal Cells Sustain Long-Term Therapeutic Efficacy Compared With Low-Dose Interleukin-2 in Systemic Lupus Erythematosus

**DOI:** 10.1093/stcltm/szad040

**Published:** 2023-06-29

This is a correction to: Zhouli Cao and others, Allogenic Umbilical Cord-Derived Mesenchymal Stromal Cells Sustain Long-Term Therapeutic Efficacy Compared With Low-Dose Interleukin-2 in Systemic Lupus Erythematosus, *Stem Cells Translational Medicine*, 2023;, szad032, https://doi.org/10.1093/stcltm/szad032

In the originally published version of this manuscript the word “Erythematosus” in the title was incorrectly published as “Erythematosu”. The publisher apologizes to readers and the authors for any inconvenience caused.

This error has been corrected.

