# Supplementary material for: Allogenic Umbilical Cord-Derived Mesenchymal Stromal Cells Sustain Long-Term Therapeutic Efficacy Compared With Low-Dose Interleukin-2 in Systemic Lupus Erythematosus
Source: Stem Cells Transl Med. 2023 Jun 6;12(7):431–43. doi: 10.1093/stcltm/szad032 (PMC10346415; doi:10.1093/stcltm/szad032)
Supplement: szad032_suppl_Supplementary_Materials [file szad032_suppl_supplementary_materials.pdf]

Supplementary Materials

Material and methods

Animals

Lupus-prone mice, the *MRL/MpJ-Fas^{lpr}* (MRL/lpr) strain, and C57BL/6 mice were used in this study. 12-week-old female MRL/lpr mice and 6- to 8-week-old C57BL/6 mice were purchased from Shanghai SLAC Laboratory Animal (Shanghai, China) were kept in specific-pathogen-free conditions in the animal center of The Affiliated Drum Tower Hospital of Nanjing University Medical School. All experimental animal protocols were approved by the Ethics Committee of the Experimental Animal Administration of this hospital (No.20191218).

Patients

All the patients met at least 4 of the 11 American College of Rheumatology criteria for SLE, with a Systemic Lupus Erythematosus Disease Activity Index 2000 (SLEDAI-2K) score of more than or equal to 8 or with at least one British Isles Lupus Assessment Group (BILAG) grade A or at least two BILAG grade B manifestations. These patients were unresponsive to previous treatment with one or more conventional immunosuppressive drugs (CYC 500 – 750 mg/m^2/month, mycophenolate mofetil 2,000 mg/day, leflunomide 20 mg/day, azathioprine 100 mg/day, Tacrolimus 2 – 4 mg/day alone or in combination for more than 6 months) or had a continuing requirement for a daily dose of ≥ 20 mg of prednisone or its equivalent.

These patients were excluded from the study if they had the following conditions: (1) contraindication to MSCs at the time of screening such as infection, including pneumonia (bacterial, virus, or fungal), pulmonary tuberculosis, hepatitis B and C, skin infection, CNS infection; (2) severe organ dysfunction such as heart failure New York Heart Association functional classification III or IV, hepatic failure, renal failure, or respiratory failure; (3) woman who were pregnant or lactating, or a woman or man who intended to initiate a pregnancy in the following 6 months.

Totally, 5 refractory lupus patients were included from the Affiliated Drum Tower Hospital of Nanjing University Medical School. The average age of these patients was 31.2 ± 5.63 and the gender ratio (female/male) was 3/2. All the subjects were treated with allogenic UC-MSCs infusion

(1 x10^6 cells/kg) and given informed consent for the collection of peripheral blood. The clinical details of the patients were shown in Table S1. The study was approved by the Ethics Committee of The Affiliated Drum Tower Hospital of Nanjing University Medical School (No.2021-662-01) and registered on ClinicalTrials.gov (Identifier: NCT01741857).

UC-MSC and low-dose IL-2 treatments

MRL/lpr mice were selected after determining whether they were at the onset of disease by detecting changes in their weekly urine protein concentration and observing whether their nasal rashes appeared. After the onset of MRL/lpr mice, we randomly divided them into four different groups, which are the UC-MSCs group, IL-2 group, UC-MSCs+IL-2 group, and negative control group. We started to give different treatments at the 16th week. Mice were sacrificed after 1-week or 4-week treatment to mimic the short- and long-term treatment, respectively.

UC-MSCs were isolated as described in our previous study[1]. The Stem Cell Center of Jiangsu Province (Beike Biotechnology) prepared the UC-MSCs. The umbilical cords were washed in PBS with added penicillin and streptomycin, and the cord blood was removed during the process. The washed cords were cut into 1-mm^2-sized pieces and then incubated in DMEM containing 10% FBS. Non-adherent cells were removed by washing. The medium was replaced every 3 days. Well-developed colonies of fibroblast-like cells were trypsinized and passage into a new flask for further expansion. At 80-85% confluence, the adherent cells were detached and cryopreserved in a solution containing 90% FBS and 10% DMSO. Thawed cells were expanded using the same protocol as described for primary expansion. Cells from passage 6 to 8 were harvested for use. The UC-MSCs were released for use only if they met specific criteria, including spindle-shaped morphology, absence of visible clumps, no cell supernatant contamination by pathogens or virus, and a cell viability greater than 92%. Immunophenotype analysis indicated that the UC-MSCs had high expression of CD73, CD105, CD90, and CD29 (>90%) and low expression of CD45, CD34, CD14, CD79, and HLA-DR (<2%). Each mouse was given 5×10^5 cells by tail vein injection once.

MRL/lpr mice were administered with 30,000 IU recombinant mouse IL-2 protein (rmIL-2) (R&D Systems, catalog# 402-ML-020, Abingdon, USA) subcutaneously daily from day 1 to day 7 for 1 week, phosphate-buffered saline (PBS) was used as negative control[2, 3].

Isolation of peripheral blood mononuclear cells (PBMCs)

In this experiment, 4 patients with SLE who met at least 4 of the 11 American College of Rheumatology criteria for SLE with a SLEDAI score of more than or equal to 8 were enrolled. Peripheral blood was collected in anti-EDTA anticoagulation tubes, centrifuged at 2000 rpm for 5 min at room temperature, and the serum was dispensed. After that, PBS was added to the initial volume of whole blood in the centrifuge tube, and the diluted whole blood was added slowly along the wall of the tube to an equal volume of Lymphoprep™ Density gradient medium for the isolation of mononuclear cells (STEMCELL Technologies, Oslo, Norway). The tube was carefully placed in a centrifuge without breaking the blood Ficoll layering interface and centrifuged at a reduced speed of 2000 rpm for 20 min. After centrifugation, the tube was seen to be divided into three layers and the cells in the middle cloud layer were carefully aspirated with a dropper and transferred to a new centrifuge tube. PBS was added to the centrifuge tube containing the cells and the tube was centrifuged at 1800rpm for 5min to collect cell precipitate. The cell precipitate was washed with PBS, resuspended in 2 ml of 10% FBS 1640 medium for counting, and set aside.

Quantitative Real-Time PCR (qRT-PCR)

Total RNA was isolated from cells by using a TRIzol reagent. Reverse transcription was performed from 2 μg of total RNA with the HiScript II Q RT SuperMix II. For qRT-PCR experiments, reactions containing cDNA, gene primers, and SYBR Green Master Mix (High ROX Premixed) were run on the StepOnePlus real-time PCR Systems (Applied Biosystems, Foster City, USA). All reagents used in the PCR experiments were purchased from Vazyme Biotech (Nanjing, China). The relative gene quantification was calculated with the $2^{-\Delta\Delta Ct}$ method.

The following primers were used (Genscript Biotech, Nanjing, China):

Gene	Species	Forward Primer (5' to 3')	Reverse Primer (5' to 3')
Il2	mouse	GCCCCAAGGGCTCAAAAATG	GCGCTTACTTTGTGCTGTCC
Gapdh	mouse	AAGGTCATCCCAGAGCTGAA	CTGCTTCACCACCTTCTTGA
IL2	human	TTACATGCCCAAGAAGGCCA	GCACTTCCTCCAGAGGTTTGA
GAPDH	human	GCACCGTCAAGGCTGAGAAC	TGGTGAAGACGCCAGTGGA

Co-culture with UC-MSCs

The UC-MSCs co-culture system was set up based on previous studies. In short, UC-MSCs were plated at a density of 5×10^4 cells/well on 24 -well or 1×10^4 cells/well on 96-well tissue culture plates, and 4 hours later 1-9 $\times 10^5$ of PBMCs from SLE patients or splenocytes from MRL/lpr mice were added into the wells. Unless stated otherwise, all cell co-culture experiments were performed in Roswell Park Memorial Institute (RPMI) 1640 Medium (MultiCell, Woonsocket, USA) supplemented with 10% fetal bovine serum, 1% streptomycin (0.025 μg/ml), 1% penicillin (0.025 U/ml), at 37°C and 5% CO_2 in a humidified atmosphere. After 12, 24, and 48 hours of co-culturing, the supernatant and cells were collected for immediate detection or stored at -80 °C.

IL-2 neutralization

For in vitro culture assays, anti-mouse IL-2 antibodies (JES6-1A12, Biolegend, San Diego, USA) were added to the culture system at the concentration of 1 ug/ml. The samples in each group were prepared in triplicate, and the experiments were repeated twice independently. To neutralize IL-2 in vivo, 0.25mg of anti-IL-2 monoclonal antibody clone S4B6-1 (BioXcell, Lebanon, USA) and the same amount of JES6-1A12 were mixed and injected intraperitoneally to mice every other day for 1 week[4].

Enzyme-Linked Immunosorbent Assay (ELISA)

Blood urea nitrogen (BUN) and serum creatinine concentrations were detected by using BUN and creatinine test kits (Jiancheng bioengineering, Nanjing, China). Serum anti-dsDNA antibodies and ANA were measured by the mouse anti-dsDNA and anti-nuclear antigens ELISA Kits (FujiFlim Wako, Osaka, Japan). IgG, IL-10, IFN-γ, TGF-β, and IL-2 in serum and cell culture supernatant were detected by mouse IgG, IL-10, IFN-γ, TGF-β, IL-2, and human IL-2 kits (Lianke BioTECH, Hangzhou, China), respectively. All the measurements were conducted following the manufacturer's instructions.

Flow cytometry and intracellular cytokine staining

To obtain single-cell suspensions, spleens and lymph nodes were mashed on ice and then filtered with the 200-mesh sieves. PBMCs were isolated by using a mouse PBMC separation solution (catlog#LDS1090, TBD science, Tianjin, China) according to the manufacturer's instructions. Cells were stimulated in a complete medium for 4h with phorbol 12-myristate 13-acetate (PMA, 50 ng/ml),

Ionomycin (Iono, 1 μg/ml) in the presence of brefeldin A (BFA, 5 μg/ml) to the accumulation of most cytokines at the Golgi Complex/Endoplasmic Reticulum. For T_H1, T_H2, and T_H17 cell staining, cells were firstly stained with FITC conjugated anti-CD4(clone# GK1.1) for 30 min in the dark at 4°C. The resulting cells were then fixed and permeabilized with a fixation/permeabilization kit (catlog# 554714) before being labeled with Percp-cy5.5 conjugated anti-mouse-IFN-γ (clone# XMG1.2), PE-conjugated anti-mouse IL-4(clone# 11B11) and APC conjugated anti-mouse-IL-17A (clone#eBio17B7) for 40 min in the dark at 4°C. For cytotoxicity analysis, cells were firstly stained with pacific blue-conjugated anti-mouse CD8 (clone# 53-6.7), followed by intracellular staining of Granzyme B (clone# QA18A28) and Perforin (S16009A). Surface staining of CD69 (clone# H1.2F3) and KLRG1 (clone# 2F1/KLRG1) were applied to access activation of $CD4^+$, $CD8^+$ T, and NK cells. For the assessment of mouse Tregs, cells were stained with FITC conjugated anti-mouse-CD4 (clone# GK1.5) and APC conjugated anti-mouse-CD25 (clone# PC61.5), followed by fixing and permeabilizing using FoxP3 / Transcription Factor Staining Buffer Set (catlog# 00-5523-00, eBioscience, San Diego, USA). Cells were washed and then stained with PE conjugated anti-mouse-FoxP3 (clone# FJK-16s). For $IL\text{-}2^+$ cell staining, cells were firstly stained with FITC conjugated anti-mouse-CD3 (clone# 17A2), APC conjugated anti-mouse-B220 (clone# RA3-6B2), Percp conjugated anti-mouse-CD11b (clone# M1/70) for 30 min in the dark at 4°C. The resulting cells were then fixed and permeabilized with a fixation/permeabilization kit (catlog# 554714) before being labeled with PE conjugated anti-mouse-IL-2 (clone# JES6-5H4) for 40 min in the dark at 4°C. For the assessment of human Tregs, cells were stained with FITC conjugated anti-human-CD4 (clone# A161A1) and APC conjugated anti-human-CD25 (clone# BC96), followed by fixing and permeabilizing using FoxP3 / Transcription Factor Staining Buffer Set (catlog# 00-5523-00, eBioscience, San Diego, USA). Cells were washed and then stained with PE-cy5.5-conjugated anti-human-FoxP3 (clone# PCH101). For ILCreg cell staining, cells were stained with FITC conjugated anti-mouse-Lin (clone#145-2C11; RB6-8C5; RA3-6B2; Ter-119; M1/70), PE conjugated anti-mouse-CD127 (clone#A7R34), and PE-Cy7 conjugated anti-mouse-CD45 (clone#30-F11). The resulting cells were then fixed and permeabilized with a fixation/permeabilization kit (catlog# 554714) before being labeled with APC conjugated anti-mouse-IL-10 (clone#JES5-16E3) for 40 min in the dark at 4°C. All antibodies and permeabilization kits were purchased from eBioscience (San Diego, USA), Biolegend (San Diego, USA), or BD Bioscience (San Diego, USA). Cells were

then washed in PBS, resuspended in 150-200 µl PBS and then analyzed using a BD LSRFortessa™ Cell Analyzer. The cytometric data were analyzed with FlowJo software (TreeStar, Ashland, USA).

Histopathological examination

After mice were sacrificed, kidneys were collected and preserved in 4% paraformaldehyde (4% PFA Fix Solution) immediately. For renal histopathology assessment, kidneys were embedded in paraffin and cut into 3-µm sections. Sections were stained with hematoxylin and eosin (H&E) for histopathology and then photographed using a microscope fitted with a digital camera (Thunder imaging system, Leica Microsystems, Germany). Histological scores of renal lesions were calculated. Mainly, the severity of glomerulonephritis, interstitial nephritis, and vessels were graded on a 1-4 scale semi-quantitatively using the grading scheme, a modification of the method to grade renal lesions.

For immunofluorescence evaluation of IgG and complement 3 (C3) deposits in the kidneys, optimal cutting temperature (OCT) compound-embedded kidneys were cut into 10-µm sections and stained with anti-C3 antibody (catlog#ab11862) and recombinant anti-mouse IgG antibody (catlog#ab190475) for morphologic analysis. All antibodies were purchased from Abcam (Boston, USA). Slides were mounted with Vectashield with DAPI (catalog# H-1200-NB, Novus Biologics, USA) and visualized using a Leica Thunder microscope. The level of glomerular fluorescence from fluorescence microscopy images was measured by ImageJ software.

Statistical analysis

For continuous data, normal distribution and homogeneity of variances were assessed by the Shapiro-Wilk W test and Levene test, respectively. When data were distributed normally, they were expressed as mean ± standard deviation (SD) and compared by the following methods. Two-tailed unpaired Student's t-test was used for comparisons between two unpaired groups, while paired t test was used between two paired groups (before/after MSCs treatment). And one-way analysis of variance (ANOVA) was for more than two groups, with Student-Newman-Keuls (SNK) q test for post hoc multiple comparisons analysis. For the multiple comparisons among different time points in the 7 lupus nephritis patients of repeated measurement data, paired t test and Bonferroni corrections were performed. When data were not normally distributed, they were expressed as

median with an interquartile range (shown in Figure S1 and S2). Wilcoxon rank sum test and Wilcoxon paired samples signed rank test was used for comparisons between two unpaired or paired groups, respectively. In addition, the Kruskal-Wallis H test with the Nemenyi test was performed for more than two groups. If not specially stated, P values less than 0.05 were considered statistically significant (*p< 0.05, **p< 0.01, ***p< 0.001, ****p< 0.0001). All analyses were performed using GraphPad Prism 8 statistical software (San Diego, USA).

Abbreviations

**Abbreviations**	**Medical Terms**
MSCs	Mesenchymal Stromal Cells
IL-2	Interleukin-2
SLE	Systemic Lupus Erythematosus
UC-MSCs	Umbilical Cord-Mesenchymal Stromal Cells
Tregs	Regulatory T Cells
PGE2	Prostaglandin E2
GvHD	Graft Versus Host Disease
hUC-MSCs	Human Umbilical Cord-Mesenchymal Stromal Cells
Foxp3	Fork Head Box P3
IL2RA	Interleukin-2 Receptor Alpha Chain
CTLA4	Cytotoxic T-Lymphocyte Associated Protein 4
TGF-B	Transforming Growth Factor Beta
hBM-MSCs	Human Bone Marrow Derived-Mesenchymal Stromal Cells
IGFBP-4	Insulin-Like Growth Factor Binding Protein-4
Teffs	Effector T Cells
ANA	Anti-Nuclear Antibodies
Anti-dsDNA Antibodies	Anti-Double Stranded DNA Antibodies
IFN-γ	Interferon Gamma
IL-10	Interleukin-10
MFI	Mean Fluorescence Intensity
ILCreg	Regulatory Innate Lymphoid Cells

OAZ	Olfactory 1/Early B Cell Factor-Associated Zinc-Finger Protein
FGF-1	Fibroblast Growth Factor-1
SLEDAI-2K	Systemic Lupus Erythematosus Disease Activity Index 2000
BILAG	British Isles Lupus Assessment Group
rmIL-2	Recombinant Mouse IL-2 Protein
PBS	Phosphate-Buffered Saline
PBMCs	Peripheral Blood Mononuclear Cells
RPMI	Roswell Park Memorial Institute
BUN	Blood Urea Nitrogen
PMA	Phorbol 12-Myristate 13-Acetate
Iono	Ionomycin
BFA	Brefeldin A
PFA	Paraformaldehyde
H&E	Hematoxylin And Eosin
C3	Complement 3
OCT	Optimal Cutting Temperature
SD	Standard Deviation
ANOVA	One-Way Analysis of Variance
SNK	Student-Newman-Keuls

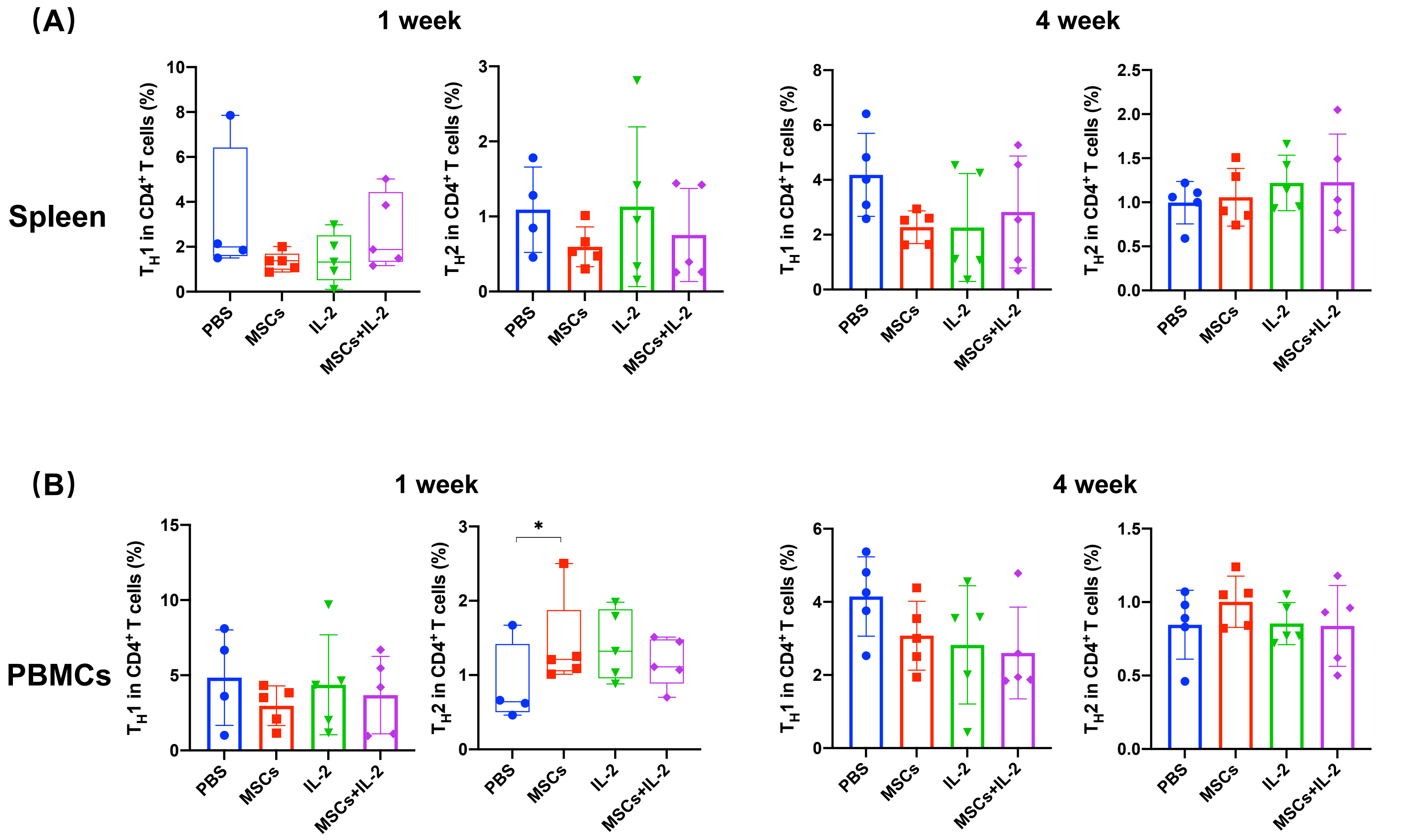

Figure S1 T_H1 and T_H2 cells in the spleen and PBMCs of MRL/lpr mice. Flow cytometric analysis of T_H1 and T_H2 cells in the spleen (A) and PBMCs (B) of MRL/lpr mice in the respective groups. All the experiments were repeated three times. PBS group in 1-week treatment, n = 4. Other groups, n = 5. *$p < 0.05$.

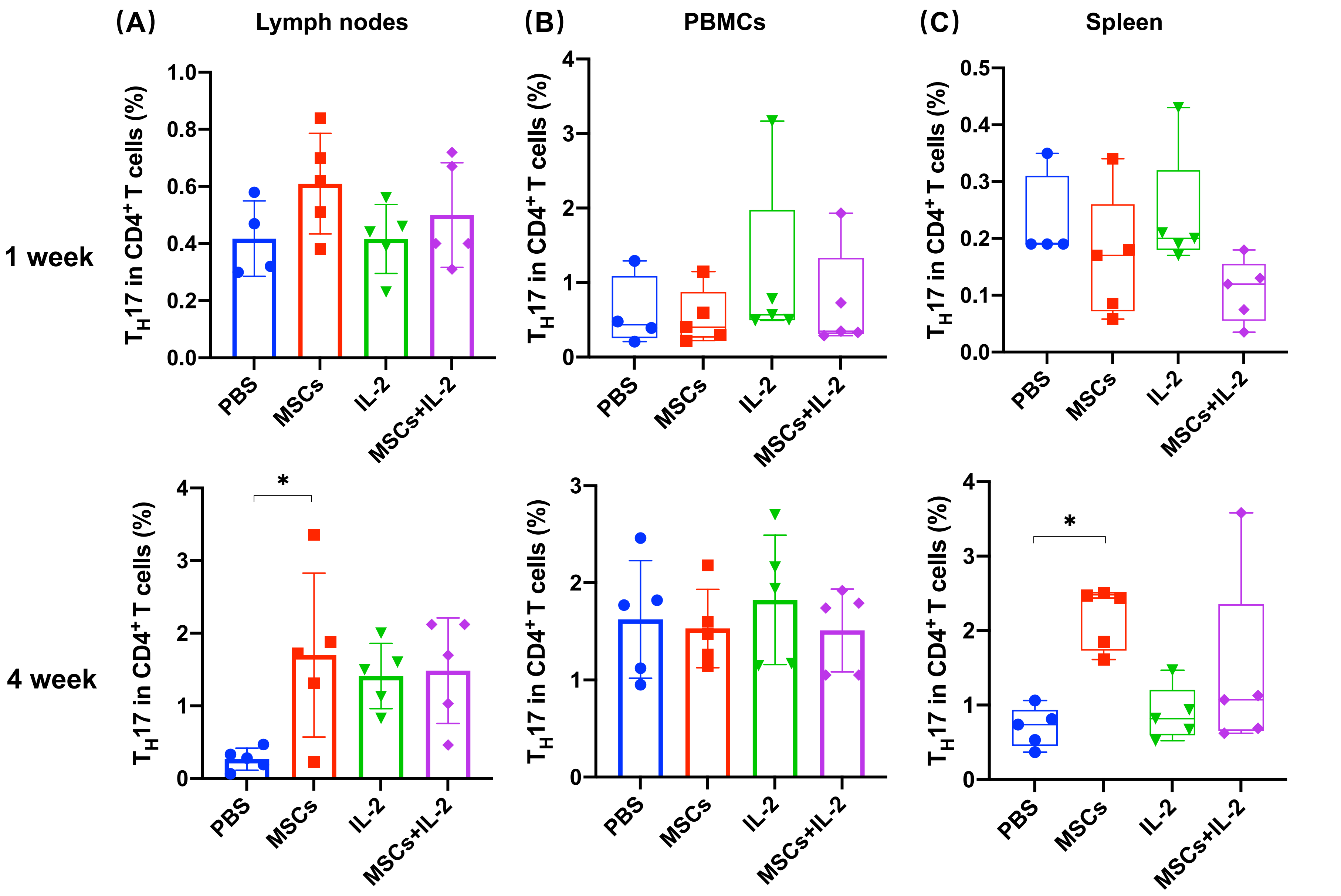

Figure S2 T_H17 in MRL/lpr mice. Flow cytometric analysis of T_H17 in the lymph nodes (A), PBMCs (B) and spleen (C) of MRL/lpr mice in the respective groups. All the experiments were repeated three times. PBS group in 1-week treatment, n = 4. Other groups, n = 5. *p< 0.05.

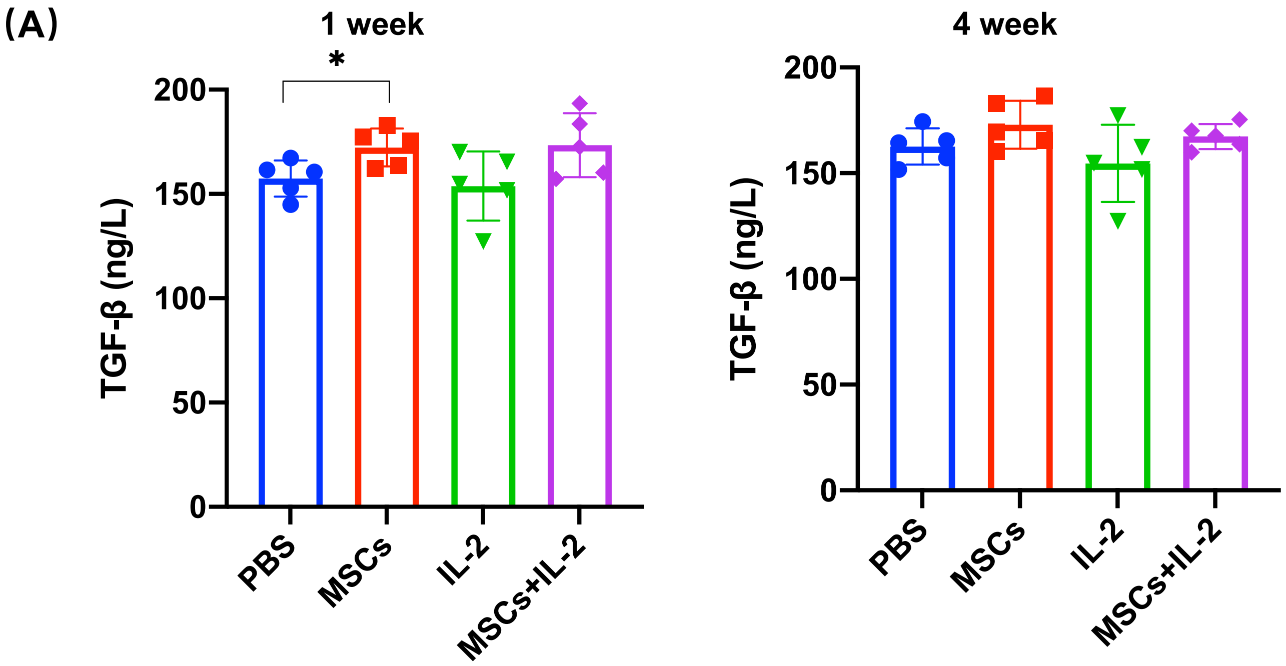

Figure S3 TGF-β in MRL/lpr mice. Serum concentrations of TGF-β in MRL/lpr mice were determined by ELISA. All the experiments were repeated three times. n = 5, per group. $*p < 0.05$.

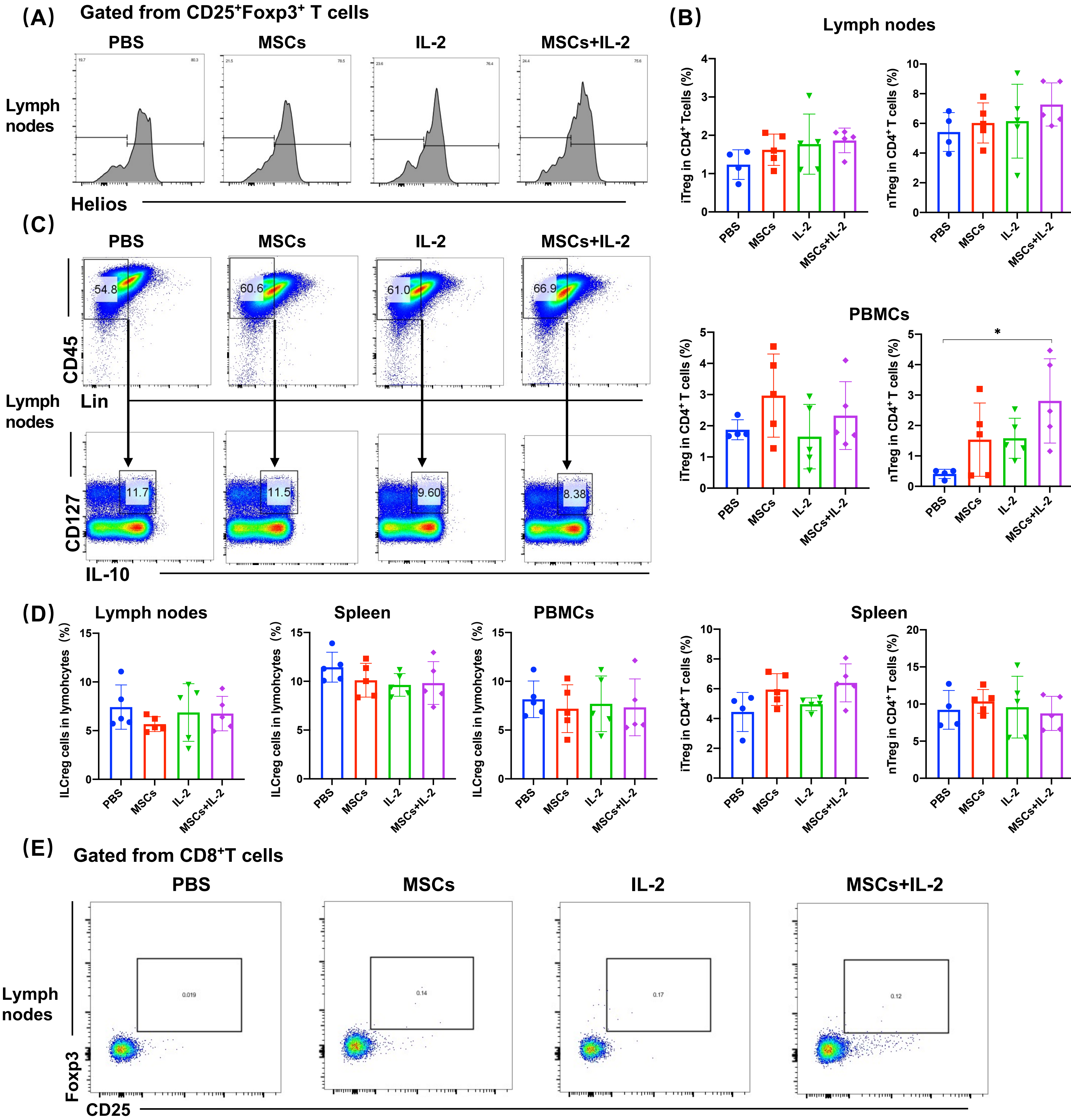

Figure S4 Influences of MSCs and low-dose IL-2 on regulatory cell subsets. (A) Flow cytometric analysis of iTregs and nTregs in the lymph nodes, PBMCs, and spleen of MRL/lpr mice in the respective groups. (A) Representative staining profiles and (B) percentages of iTregs and nTregs were shown. (C-D) Flow cytometric analysis of ILCregs (Lin$^-$CD45$^+$IL-10$^+$CD127$^+$) in the lymph nodes, PBMCs, and spleen of MRL/lpr mice in the respective groups. (C) Representative staining profiles and (D) percentages of ILCregs were shown. (E) Flow cytometric analysis of CD8$^+$Tregs in the lymph nodes of MRL/lpr mice in the respective groups. (E) Representative staining profiles were shown. All the experiments were repeated three times. PBS group (A-B), n = 4. Other groups, n = 5. *$p < 0.05$, **$p < 0.01$,

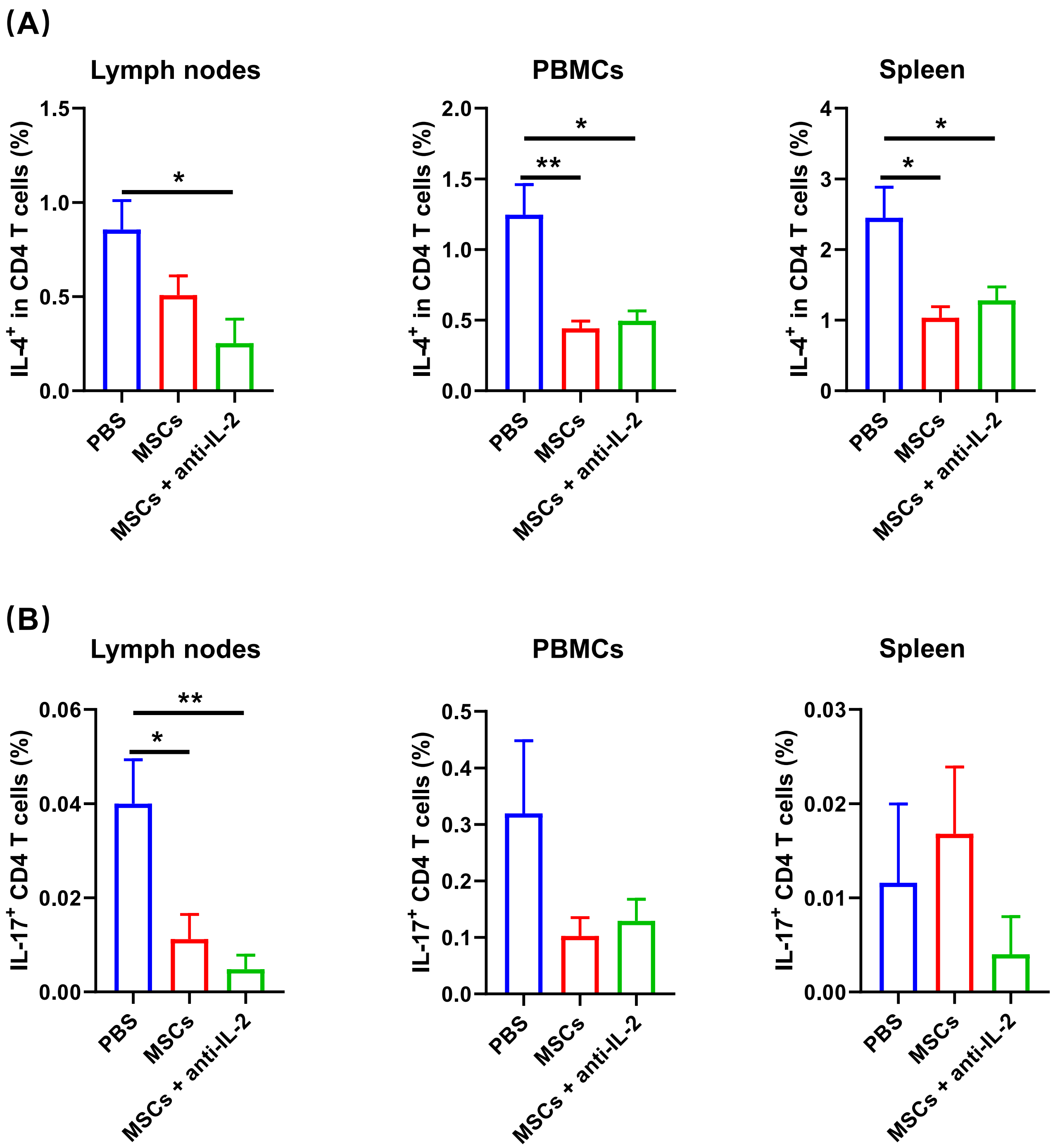

Figure S5 Neutrolization of IL-2 did not alter the modulation of T_H2 and T_H17 cells by MSCs. After receiving MSCs, MRL/lpr mice were treated with IL-2 neutralizing antibodies or istotype IgG. Mice received PBS were set as control. T_H2 (A) and T_H17 cells (B) of lymph nodes, PBMCs and spleens collected from the respective groups were analyzed by flow cytometry. All the experiments were repeated three times. n = 5. *p< 0.05.

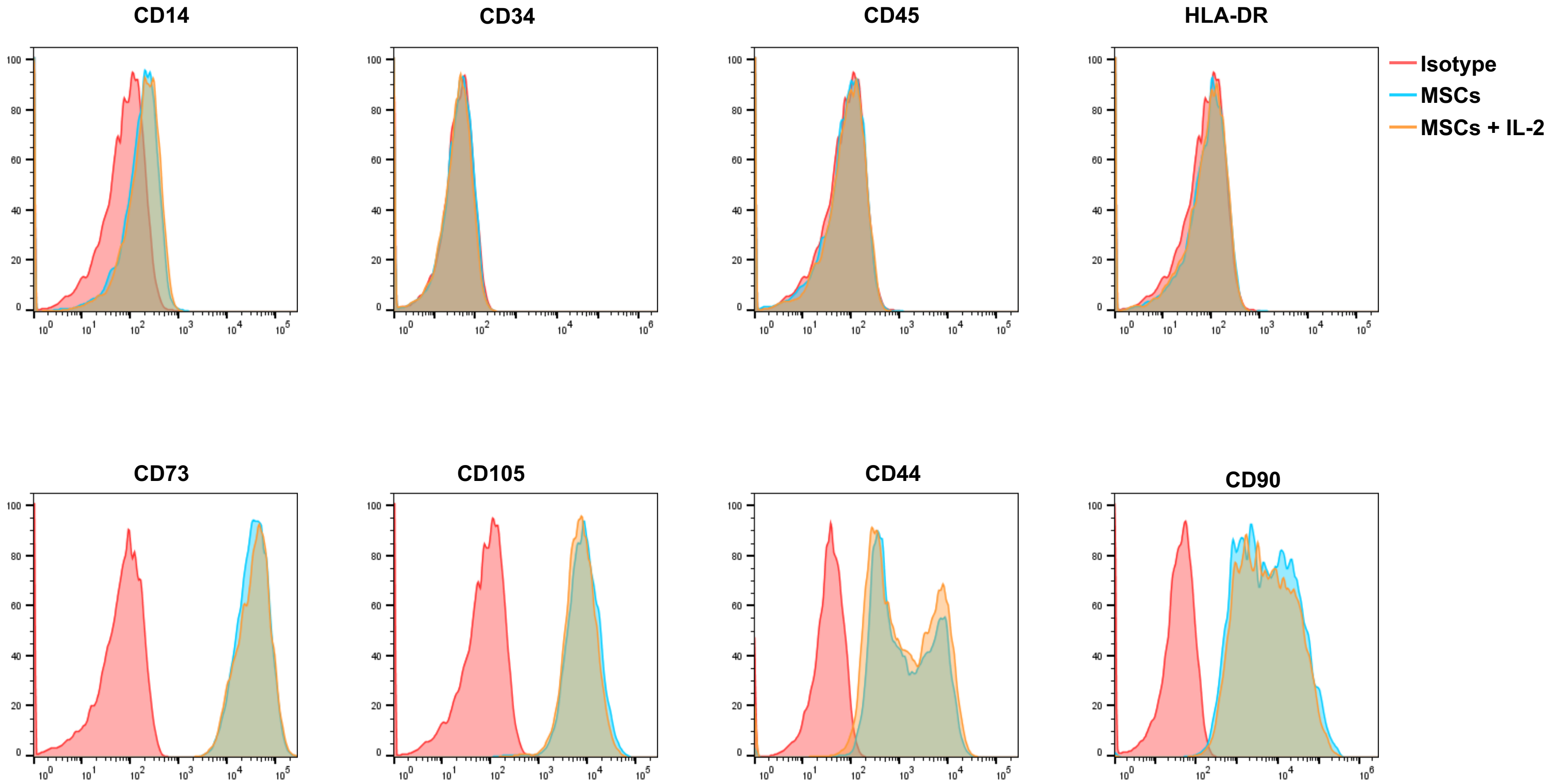

Figure S6 IL-2 did not change the phenotype of MSCs. MSCs were cultured in the presence of mouse recombinant IL-2 for 24 hours, followed by staining with antibodies against MSC-specific surface markers CD73, CD105, CD44, CD90, and negative cocktail (CD14, CD34, CD45, and HLA-DR) and analyzed by flow cytometry. The representative data were shown. All the experiments were repeated three times.

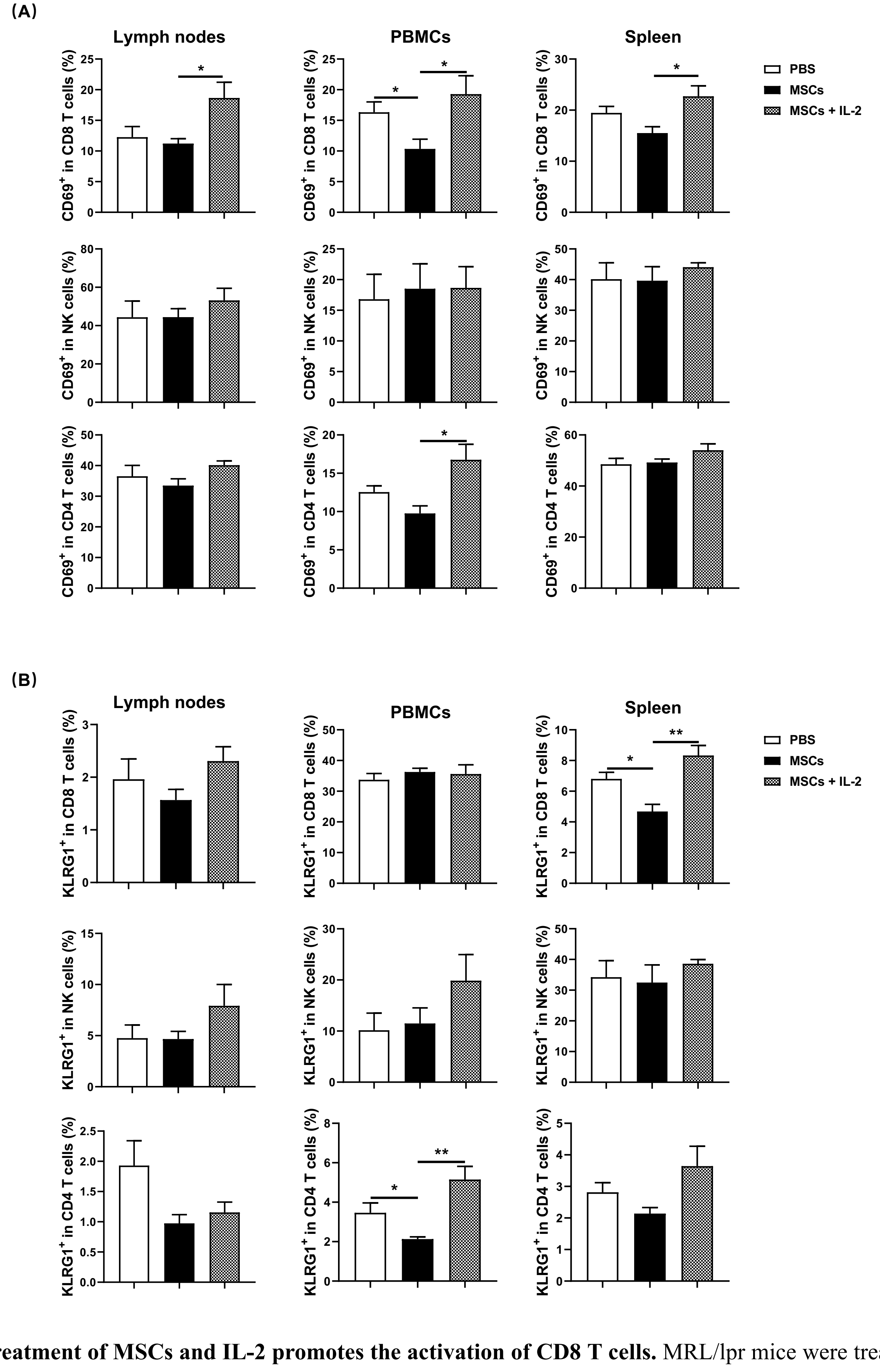

Figure S7 Combined treatment of MSCs and IL-2 promotes the activation of CD8 T cells. MRL/lpr mice were treated as depicted in Figure 1 and sacrificed 7 days after the treatment. Expression of CD69 (A) and KLRG1 (B) by $CD8^{+}$, $CD4^{+}$ T, and NK cells of lymph nodes, PBMCs, and spleen collected from the respective groups were determined by flow cytometry. All the experiments were repeated three times. n = 5. *$p< 0.05$, **$p< 0.01$.

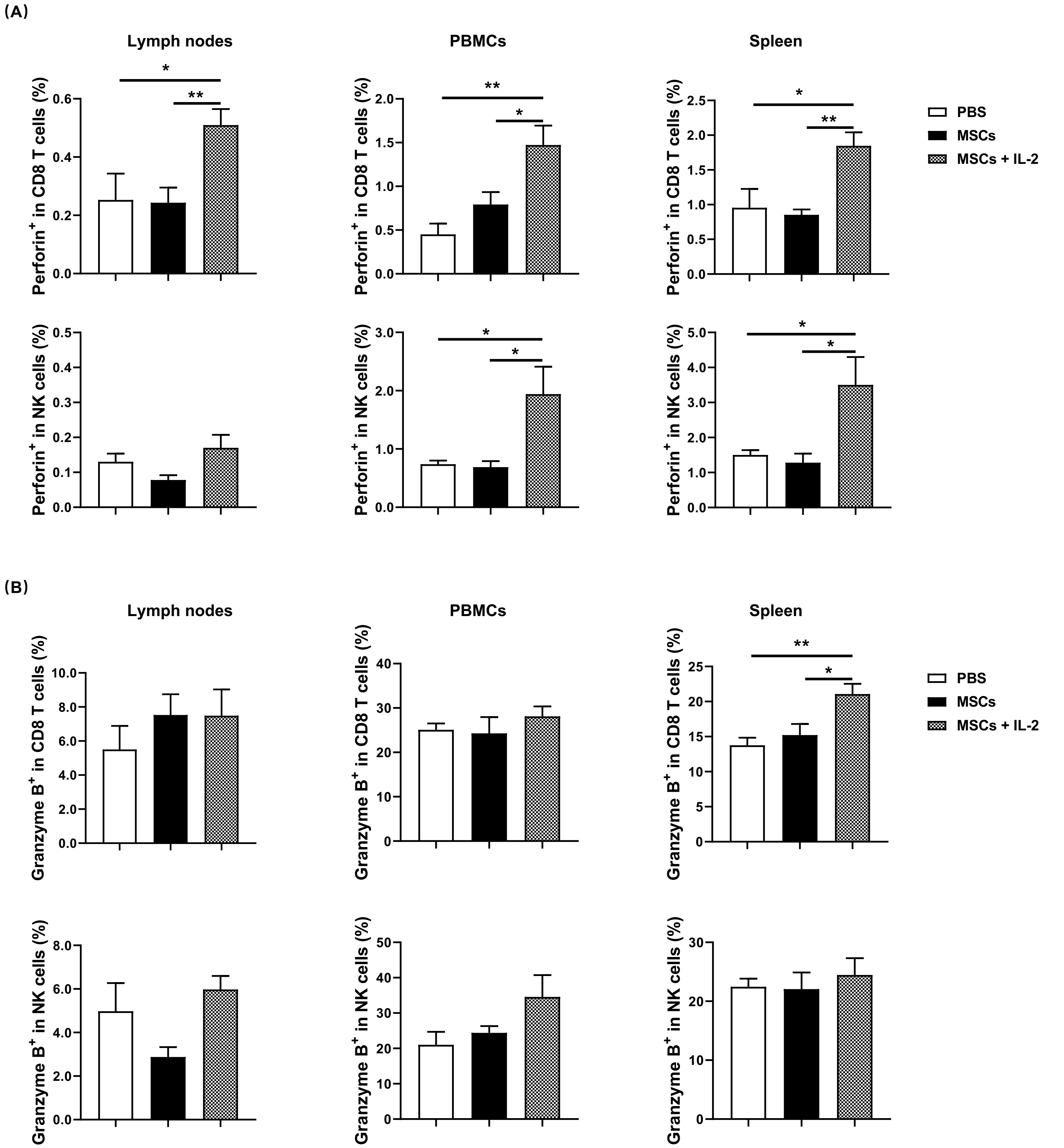

Figure S8 Perforin production by CD8 T and NK cells were enhanced by MSCs and IL-2 combined treatment. MRL/lpr mice were treated as depicted in Figure 1 and sacrificed 7 days after the treatment. Cells from lymph nodes, PBMCs and spleens of the respective groups were stimulated with PMA and Ionomycin in the presence of Brefeldin A for 4 hours. Then perforin (A) and granzyme B (B) production was determined by intracellular staining. All the experiments were repeated three times. n = 5. $^*p < 0.05$, $^{**}p < 0.01$.

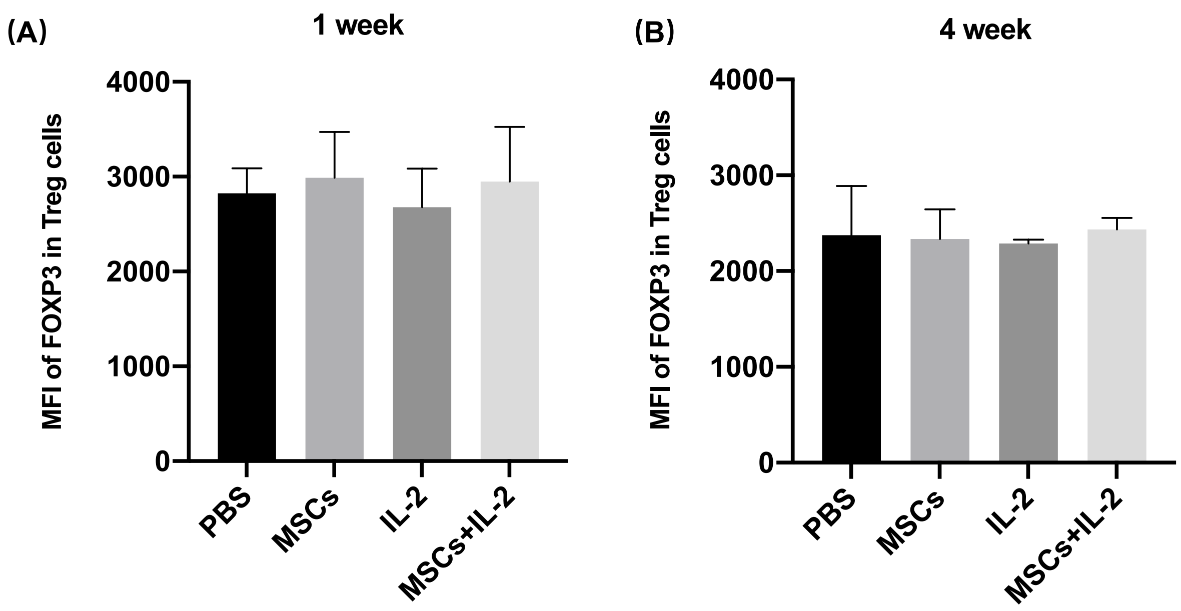

Figure S9. Expression of FOXP3 by Treg cells. FOXP3 expressed by Tregs from lymph nodes was determined by FACS and the Mean Fluorescence Intensity (MFI) was calculated. All the experiments were repeated three times. PBS group in 1-week (A), n = 4. Other groups, n = 5. *$p < 0.05$, **$p < 0.01$, ***$p < 0.001$, ****$p < 0.0001$. w, week.

Supplementary Table 1. Baseline clinical characteristics and medications of SLE patients receiving UC-MSCs treatment

Patient number	age/gender	Disease duration (months)	Clinical manifestation	Treatment before UC-MSCs treatment
1	32/F	48	LN, AL, LC, anti-dsDNA	Pred +LEF +HCQ
2	31/M	96	LN, R, LC	Pred +HCQ +Tacrolimus
3	34/F	84	A, LN, R, AL, M, LC	Pred +HCQ +MMF +Tacrolimus
4	22/M	24	V, A, LN, R, AL, LC, anti-dsDNA, T, L	Pred +HCQ +MMF
5	37/F	96	LN, R, LC, anti-dsDNA, F, L	Pred +HCQ +MMF

A, arthralgia; LN, lupus nephritis; R, Rash; AL, Alopecia; LC, Low complement; anti-dsDNA, anti-double strand DNA antibodies; F, febrile; T, Thrombocytopenia; L, Leukopenia;

Pred, prednisone; CYC, Cyclophosphamide; LEF, Leflunomide; HCQ, Hydroxychloroquine; MMF, Mycophenolate Mofetil; mo, month.

Reference

1. Yuan X, Qin X, Wang D, et al. Mesenchymal Stem Cell Therapy Induces Flt3l and Cd1c(+) Dendritic Cells in Systemic Lupus Erythematosus Patients. *Nature communications* 2019;10(1):2498-98.
2. He J, Zhang X, Wei Y, et al. Low-Dose Interleukin-2 Treatment Selectively Modulates Cd4(+) T Cell Subsets in Patients with Systemic Lupus Erythematosus. *Nat Med* 2016;22(9):991-3.
3. Taylor EB, Sasser JM, Maeda KJ, et al. Expansion of Regulatory T Cells Using Low-Dose Interleukin-2 Attenuates Hypertension in an Experimental Model of Systemic Lupus Erythematosus. *Am J Physiol Renal Physiol* 2019;317(5):F1274-f84.
4. McNally A, Hill GR, Sparwasser T, et al. Cd4+Cd25+ Regulatory T Cells Control Cd8+ T-Cell Effector Differentiation by Modulating Il-2 Homeostasis. *Proceedings of the National Academy of Sciences* 2011;108(18):7529-34.
